# Discovery of a New Class of Aminoacyl Radical Enzymes Expands Nature’s Known Radical Chemistry

**DOI:** 10.1021/jacs.4c10348

**Published:** 2024-10-11

**Authors:** Beverly Fu, Hao Yang, Duncan J. Kountz, Maike N. Lundahl, Harry R. Beller, William E. Broderick, Joan B. Broderick, Brian H. Hoffman, Emily P. Balskus

**Affiliations:** † Department of Chemistry and Chemical Biology, Harvard University, Cambridge, Massachusetts 02138, United States; ‡ Department of Chemistry, Northwestern University, Evanston, Illinois 60208, United States; § Department of Chemistry and Biochemistry, Montana State University, Bozeman, Montana 59717, United States; ∥ Lawrence Berkeley National Laboratory, Berkeley, California 94720, United States; ⊥ Department of Chemical Engineering and Applied Chemistry, University of Toronto, Toronto, ON M5S 3E5, Canada; # Howard Hughes Medical Institute, Harvard University, Cambridge, Massachusetts 02138, United States

## Abstract

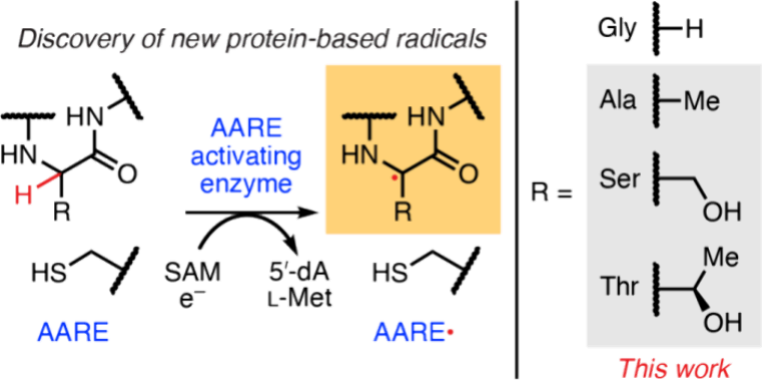

Radical enzymes, including the evolutionarily ancient glycyl radical enzyme (GRE) family, catalyze chemically challenging reactions that are involved in a myriad of important biological processes. All GREs possess an essential, conserved backbone glycine that forms a stable, catalytically essential α-carbon radical. Through close examination of the GRE family, we unexpectedly identified hundreds of noncanonical GRE homologs that encode either an alanine, serine, or threonine in place of the catalytic glycine residue. Contrary to a long-standing belief, we experimentally demonstrate that these aminoacyl radical enzymes (AAREs) form stable α-carbon radicals on the three cognate residues when activated by partner activating enzymes. The previously unrecognized AAREs are widespread in microbial genomes, highlighting their biological importance and potential for exhibiting new reactivity. Collectively, these studies expand the known radical chemistry of living systems while raising questions about the evolutionary emergence of the AAREs.

## Introduction

**Figure 1:**
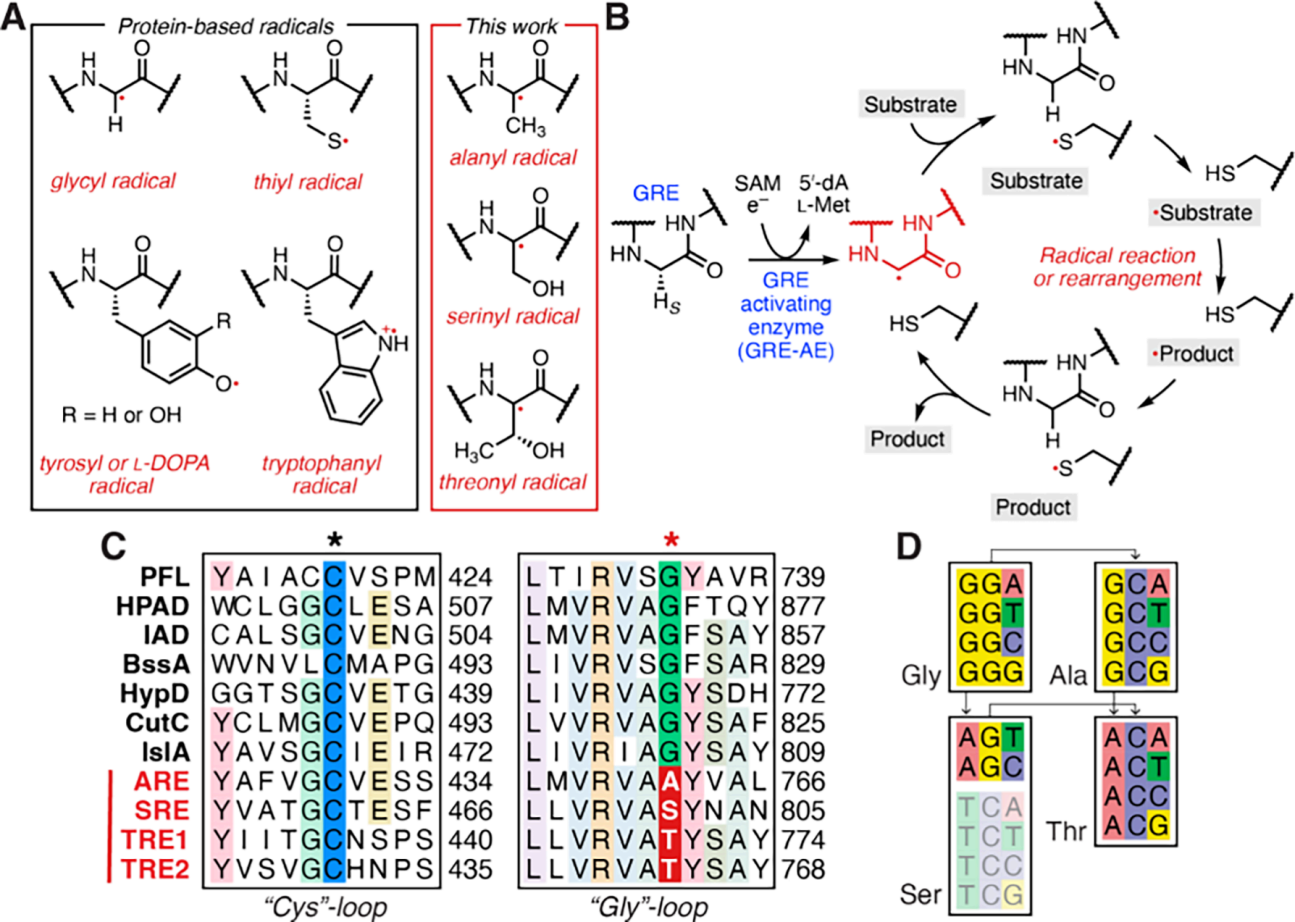
Identification of GRE homologs lacking the catalytic Gly. (A) Known amino acid–based protein radicals reside on l-Tyr, l-Cys, Gly, l-Trp, and l-DOPA. Newly characterized radicals reside on l-Ala, l-Ser, and l-Thr. (B) In the widely accepted proposed GRE activation mechanism, 5′-dA• abstracts the *pro-S* hydrogen atom of Gly to generate a glycyl radical highlighted in red. The glycyl radical generates a proposed thiyl radical intermediate that mediates the reaction with the substrate. (C) Representative multiple-sequence alignment of AAREs compared to a subset of biochemically characterized GREs. Although there is general conservation of the Cys and Gly fingerprint motif, the catalytic Gly site (red asterisk) is substituted with an Ala, Ser, or Thr. (D) Codons that encode Gly, Ala, Ser, and Thr are single-nucleotide point mutations away from each other.

Free radicals are high-energy chemical species with an unpaired electron, most commonly associated with reactive oxygen species in biological systems.^1^ Uncontrolled generation of free radicals within cells can cause oxidative stress and contribute to human disease.^1,2^ In contrast, Nature has evolved radical enzymes that harness reactive species for catalysis. These enzymes are ubiquitous in life and use single-electron chemistry to catalyze enzymatic transformations inaccessible via two-electron mechanisms.^3,4^ Many of these reactions are critical for primary metabolism, such as converting ribonucleotides to deoxyribonucleotides,^5^ anaerobic glucose metabolism,^6^ cofactor biosynthesis,^7^ and methanogenesis.^8^ Radical enzymes employ two broad categories of radical intermediates: those generated on organic or metal-based cofactors and those post-translationally installed on the polypeptide chain itself (protein-based radicals).^9^ The most prevalent radical intermediate in enzymes is the carbon-centered 5′-deoxyadenosyl radical (5′-dA•) employed by all members of the canonical radical *S*-adenosyl-l-methionine (rSAM) superfamily.^10−16^ As for protein-based radicals, a variety of stable and transient amino acid radical intermediates and cofactors have been characterized, with the vast majority residing on the proteinogenic amino acids l-Tyr (O•), l-Cys (S•), Gly (Cα•), and l-Trp (π-cation•) (Figure 1A).^17^ More recently, 3,4-dihydroxyphenylalanine (l-DOPA) in class Ie ribonucleotide reductase (RNR), derived from post-translational modification of tyrosine, was found to harbor a catalytically competent l-DOPA radical.^18,19^ Transient Cα radical intermediates have been observed on l-Ile and l-Val residues of peptide substrates during radical epimerization.^20^ In the absence of substrate, radical enzymes have also been reported to form nonproductive l-His (C2•) or l-Val (Cβ•) radicals on their own polypeptide chain.^21,22^ Notably, the characterized protein-based radicals in known radical enzymes are limited to a small subset of amino acids.

The glycyl radical enzyme (GRE) family is a widespread group of radical enzymes found in anoxic environments such as the human gut microbiome. These evolutionarily ancient enzymes,^14^ which include pyruvate formate-lyase (PFL) and anaerobic class III RNR (NrdD),^23,24^ use a conserved, O_2_-sensitive Gly α-carbon-centered radical to initiate a range of challenging chemical transformations. Discovery of new GREs has accelerated in the past decade, revealing a diversity of C–C, C–O, C–N, and C–S bond-breaking and bond-forming transformations.^24−30^ Stable under strictly anoxic conditions, the critical glycyl radical^31,32^ is post-translationally installed on the protein backbone by a dedicated partner activating enzyme (GRE-AE) belonging to the rSAM superfamily.^10−14^ Using an [Fe_4_S_4_]^+^ cluster, the GRE-AE reductively cleaves SAM,^33,34^ generating a reactive 5′-dA• species^35,36^ that abstracts the *pro*-*S* hydrogen atom (H-atom)^37^ from the conserved GRE backbone Gly (Figure 1B).^15,38,39^ In the proposed catalytic cycle of all GREs, the Gly radical first abstracts an H-atom from a nearby conserved Cys, forming a transient thiyl radical that reacts with the substrate via H-atom abstraction or electron transfer. Further reaction of the substrate-based radical generates a product-centered radical that reabstracts an H-atom from Cys, providing product and regenerating the protein-based thiyl radical. Finally, the thiyl radical reabstracts an H-atom from Gly, restoring the glycyl radical.

The glycyl radical serves as the resting state between each round of substrate turnover in a GRE. The utility of this particular radical intermediate in catalysis is thought to be linked to its stability and conformational flexibility; the captodative effect arising from the adjacent electron-withdrawing amide carbonyl and electron-donating amide nitrogen contributes an additional 34.8 kJ mol^–1^ stabilization to the glycyl radical.^40−43^ Since the initial discovery and characterization of the glycyl radical in PFL via electron paramagnetic resonance (EPR) spectroscopy,^31,32^ this cofactor has been found to be essential in all GRE-catalyzed reactions investigated to date.^24−30^ Moreover, other radical enzymes not part of the GRE family are proposed to use glycyl radical intermediates generated by alternative mechanisms (Figure S1).^44−49^ Although similar α-carbon-centered aminoacyl radicals could theoretically be generated from other proteinogenic amino acids, computational modeling suggests these α-carbon radicals are less stable, as the electronic stabilization may be outweighed by the repulsive steric interaction between the amino acid side chain and backbone amide.^40,50^ Indeed, the glycyl radical is the only α-carbon-centered amino acid radical cofactor currently known in biochemistry.

Here, we report the unexpected discovery of nonglycyl radical-containing relatives of GREs. Protein sequence analysis and structure prediction indicate that these enzymes are homologous to GREs but have evolved to replace the critical glycine with either Ala, Ser, or Thr. EPR spectroscopy shows that heterologously expressed aminoacyl radical enzymes (AAREs), like GREs, are post-translationally modified by cognate rSAM-activating enzymes to generate α-carbon radicals but on Ala, Ser, or Thr instead of Gly. This work reveals previously unrecognized radical-based post-translational modifications, thus challenging previous assumptions about the GRE superfamily and viable protein-based radicals in enzymatic catalysis.

## Results

### Bioinformatic Analyses Identify Noncanonical GRE Homologs

Prior work, particularly with the first family member discovered, PFL, has established principles thought to be universal to the GRE family. GREs are readily identified bioinformatically by a highly conserved C-terminal GRE fingerprint motif containing the catalytic Gly (RVX**G**[FWY])^51^ and a conserved Cys near the middle of the protein corresponding to the site of the proposed thiyl radical. The loops harboring these two residues are buried within GRE structures.^6^ In addition, GREs are typically encoded near their partner GRE-activating enzymes (GRE-AEs) in genomes. One underinvestigated sequence repository is that of metagenome-assembled genomes (MAGs), which includes sequences from uncultured microbes. In examining sewage-derived metagenomes,^25^ we noticed a few peculiar GRE-like protein-encoding sequences that lacked a Gly in the GRE fingerprint region and instead encoded a Thr residue. Each sequence still preserved the catalytic Cys, suggesting that these identified proteins may be threonyl radical enzymes (TREs). Further examination of the raw sequencing data confirmed that there was no assembly error.

To identify additional putative TREs, we manually explored all sequences in the InterPro family IPR004184 (PFL domain), which currently encompasses >25,000 GREs, excluding the anaerobic class III RNRs. Upon generating a multiple-sequence alignment (MSA),^52^ we found 14 TRE sequences that contained the Gly-to-Thr substitution. Surprisingly, we also identified six sequences in which catalytic Gly is replaced by Ala (AREs) and six sequences containing Ser at this position (SREs) (Figure 1C). Iterative Basic Local Alignment Search Tool (BLAST) searches^53^ against the National Center for Biotechnology Information (NCBI) nonredundant protein database expanded our list to encompass 17 AREs (Table S1), 21 SREs (Table S2), and 71 TREs (Table S3), for a total of 109 unique AAREs. Intriguingly, most of the sequences were from MAGs from the human gut microbiome. When we performed this same search with anaerobic class III RNRs (IPR012833), we detected only the canonical Gly-encoding RNRs.

To verify that these substitutions were not sequencing errors, we considered how these residues are encoded in relation to Gly. A single G → C nucleotide change in the second position of any Gly-encoding codon would lead to an Ala substitution. Moreover, although Ser is encoded by six codons, the SREs we identified use codons derived from only a single G → A nucleotide change in the first position of two Gly-encoding codons. Interestingly, two sequential point mutations are required for the Gly-to-Thr substitution to occur, potentially through Ala- or Ser-encoding codon intermediates (Figure 1D). Taken altogether, the fact that multiple distinct AAREs were identified and that the Gly-to-Thr substitution is not a simple point mutation suggest that the AAREs are authentic GRE homologs. Further evidence supporting these assignments is that all AAREs are encoded near a putative AARE-AE that possesses the conserved [Fe_4_S_4_]-cluster-binding sequence features of an rSAM enzyme. While the AREs and SREs, like GREs, are each encoded next to a cognate AARE-AE, two TREs are generally encoded adjacent to a single TRE-AE, which is an atypical arrangement (Figure S2).

To assess whether AAREs could be post-translationally modified to form protein-based radical cofactors, we focused further computational and experimental analyses on four enzymes: the ARE from *Desulfoscipio geothermicus* DSM 3669 (*Dg*ARE), the SRE from *Dethiosulfatibacter aminovorans* DSM 17477 (*Da*SRE), and two TREs from *Flavonifractor plautii* 2789STDY5834892 (*Fp*TRE1 and *Fp*TRE2). We chose these organisms because they are cultured isolates and their whole genomes are sequenced.^54^

We first investigated whether the predicted structures of the AAREs retained core features of the GREs. Consistent with their sequence similarity, AlphaFold2^55^ predicts that the AARE monomers form a barrel composed of 10 alternating α-, β-strands, analogous to the X-ray crystal structure of *Escherichia coli* PFL (PDB: 2PFL) (Figure S3A).^6^ Multimer docking predicts that AREs and TREs can form homodimers at interfaces similar to those of crystallized GREs, whereas models for *Da*SRE and other SRE homologs predict a distinct dimer. Examination of the relative positions of the AARE “Gly”- and “Cys”-loops and comparison to structurally characterized GREs revealed that the orientations of key structural loops and the catalytic Cys are consistent across all structures (Figure S3B). Moreover, the residue proposed to harbor the initial radical (Ala, Ser, Thr, or Gly) is positioned near Cys, suggesting that H-atom transfer between the pair of residues is possible in the AAREs.

Next, we examined the sequences of the AARE-AEs to determine whether they resemble those of the GRE-AEs. The AARE-AEs overwhelmingly preserve the key Cys residues predicted to bind [Fe_4_S_4_] clusters in both sequence (Figure S4AB) and spatial (Figure S4C) orientation. The predicted AARE-AE structures are similar to X-ray crystal structures of other rSAM enzymes, including PFL-AE (PDB: 3CB8),^56^ the only structurally characterized GRE-AE. Cys residues predicted to ligate the SAM-binding [Fe_4_S_4_] cluster (CX_3_CX_2_C) are positioned in the same region. Altogether, these bioinformatic studies indicate that the AARE systems possess all of the factors required for activity.

### Properties of AAREs and AARE-AEs Are Analogous to Those of GREs and GRE-AEs

We hypothesized that the many AAREs identified bioinformatically undergo post-translational modification to generate alternative protein-based radicals. To explore this hypothesis, we set out to biochemically characterize the representative AAREs. We obtained codon-optimized genes encoding AARE and AARE-AE pairs and cloned, expressed, and purified them using established methods for GREs (Figure S5AB). Size-exclusion chromatography (SEC) of the purified AAREs (Figure S5C) revealed mixed oligomeric states, including homodimers, similar to previously characterized GREs.^29,57−60^ We also examined whether AARE heterodimer formation is possible in the case of the two TREs from *F. plautii*, which are encoded near only one TRE-AE. To test this potential, we passed lysates of coexpressed N-His_6_-*Fp*TRE1 and N-Strep-*Fp*TRE2 through a Ni-NTA affinity column. Assessment of SDS-PAGE gel and Western blot with an α-Strep antibody (Figure S5DE) indicates a weak TRE interaction, as *Fp*TRE2 eluted across a broad range of imidazole concentrations. All in all, there is a possibility for TRE1–2 heterodimer formation, although the relevance to the biochemical functions of these proteins is unknown.

To test for radical installation on AAREs, we needed to access cognate activating enzymes. Characterization of the cofactors and activity of these AARE-AEs confirmed that they are rSAM enzymes. Following anaerobic purification, we obtained brown protein solutions with UV–vis absorption spectra displaying a shoulder around 410 nm that disappeared upon reduction with sodium dithionite (NaDT), indicative of redox-active [Fe_4_S_4_] clusters (Figure S5FGH). To characterize the [FeS] clusters in *Fp*TRE-AE, we initially turned to X-band (9.373 GHz) EPR spectroscopy, which revealed the signal of a [Fe_4_S_4_]^1+^ cluster that changes only slightly with the addition of SAM because of the contribution from the auxiliary clusters (Figure S6A). The majority of GRE-AEs possess additional or auxiliary [Fe_4_S_4_]^1+^ clusters of unknown function. These spectra are similar to those previously reported for *Oa*CutD, a ferredoxin-domain containing GRE-AE.^58^

Incubation of *Fp*TRE-AE with NaDT and SAM definitively demonstrated that the rSAM [Fe_4_S_4_]^1+^ cluster of *Fp*TRE-AE carries out reductive cleavage of SAM. We observed time-dependent production of 5′-dA, as detected by ultrahigh-performance liquid chromatography–tandem mass spectrometry (UHPLC–MS/MS) (Figure S6B). This suggests that *Fp*TRE-AE is a canonical radical SAM enzyme and is catalytically active. The alternative SAM cleavage product *S*-adenosyl-l-homocysteine was not detected, and the detected methylthioadenosine product likely arose from enzyme-independent SAM hydrolysis. Altogether, the results of these experiments are consistent with *Fp*TRE-AE and other AARE-AEs being canonical rSAM enzymes.

### AAREs Harbor Stable, Protein-Based Radicals

**Figure 2:**
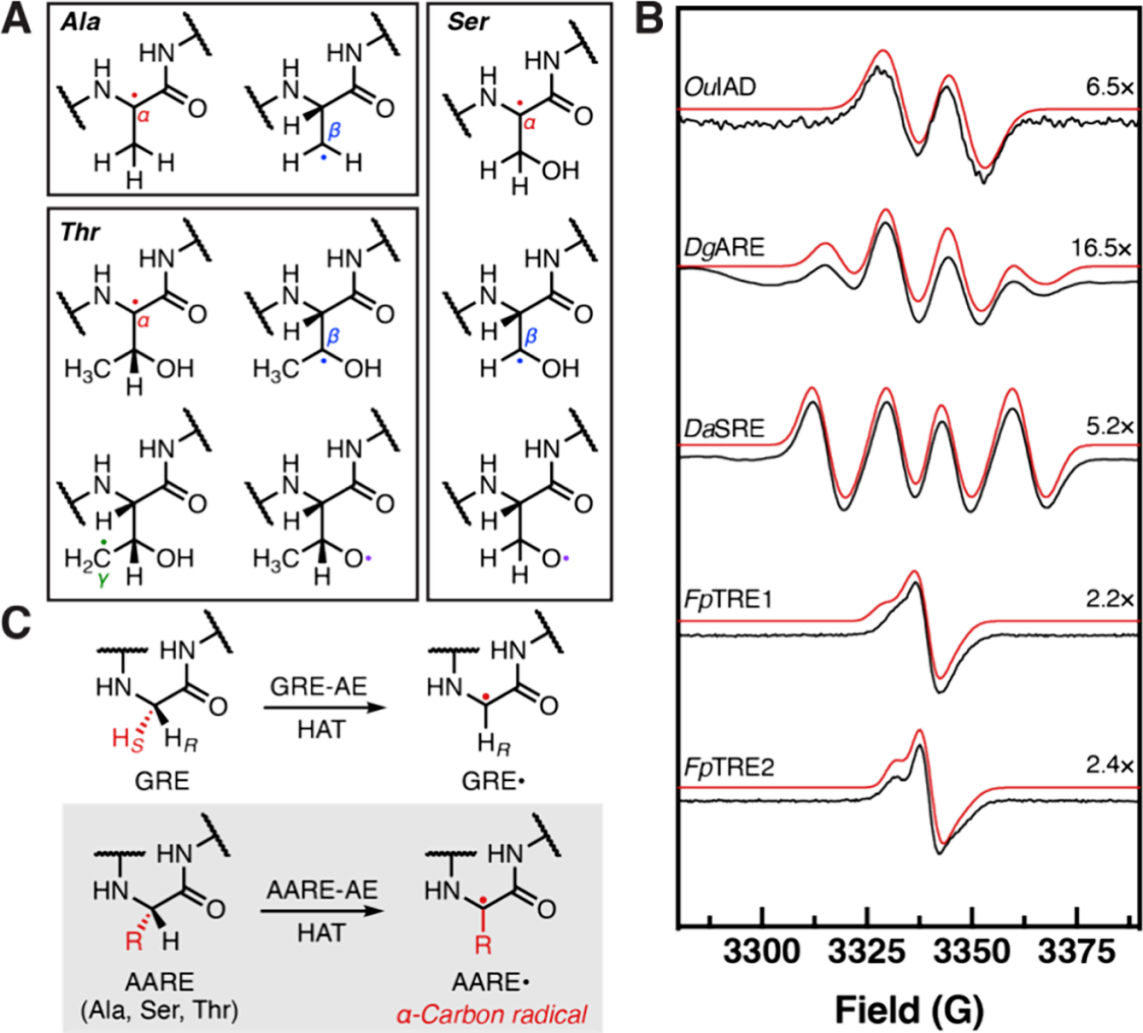
AARE activation generates alanyl, serinyl, and threonyl radicals. (A) Structures of potential radicals considered. (B) Experimental (black) and simulated (red) continuous-wave (CW) X-band EPR spectra of *Ou*IAD (Gly•), *Dg*ARE (Ala•), *Da*SRE (Ser•), *Fp*TRE1 (Thr•), and *Fp*TRE2 (Thr•) (77 K); simulations are discussed in Table S6. Spectra are scaled to better distinguish features. (C) Scheme of general radical installation on the AAREs by the AARE-AEs, compared to GRE-AEs.

To assess whether AAREs could be activated by AARE-AEs and to characterize the structures of any resulting protein-based radicals, we used X-band EPR spectroscopy. The EPR spectra of glycyl radicals exhibit a characteristic doublet (*g* = 2.0038; *A* = 39 MHz) due to hyperfine coupling of the unpaired electron to the remaining α-proton.^32^ AREs, SREs, and TREs could conceivably generate related backbone-based radicals if their activation involves H-atom abstraction at the amino acid α-carbon. However, additional possible sites of radical installation include the β-carbon, γ-carbon, and oxygen centers of the non-Gly side chains (Figure 2A).

We first tested whether *Dg*ARE could be recognized and activated by *Dg*ARE-AE. Incubating the two enzymes together with SAM and NaDT for 2 h generated a protein-based radical exhibiting a quartet ^1^H hyperfine-coupling pattern with intensity ratios of roughly 1:3:3:1 rather than the characteristic doublet of GREs^23,26−30,32,58,61,62^ like indoleacetate decarboxylase from *Olsenella uli* (*Ou*IAD), which we included as a positive control (Figure 2B).^57^ In other words, a distinct protein-based radical is generated on *Dg*ARE. This EPR spectrum is best simulated with *g* = 1.999, 2.004, 2.007 and three roughly equivalent protons with isotropic coupling, *A*_iso_ = ∼60 MHz. The quartet hyperfine-splitting pattern is as expected for a 2pπ α-carbon Ala radical whose three methyl-group β-protons, which have essentially isotropic hyperfine couplings,^63^ have become equivalent through rotational averaging.^64^ This spectrum does not correspond to that of an Ala β-carbon 2pπ radical formed by H-atom abstraction from CH_3_, as that would exhibit highly anisotropic couplings to the two remaining protons, along with an isotropic coupling to the α-proton (Figure 2A). At lower temperatures (4 and 10 K), the lines of the quartet broaden (Figure S7A), which indicates that the rotation of the methyl group slows. Spin quantitation indicated that 4.5% of *Dg*ARE monomers harbored this radical.

Incubation of *Da*SRE with *Da*SRE-AE generated a protein-based *Da*SRE radical with a four-line hyperfine pattern with an intensity ratio of roughly 1:1:1:1, distinct from those of both the alanyl and the glycyl radical spectra (Figure 2B). The 1:1:1:1 hyperfine-splitting pattern is assignable to isotropic hyperfine couplings to two protons, with one coupling twice as large as the other. The EPR spectrum of the *Da*SRE radical is best simulated with *g* = 2.004, 2.005, 2.006; *A*_iso_(^1^H_a_) = 60 MHz, and *A*_iso_(^1^H_b_) = 30 MHz, which is consistent with an α-carbon-centered Ser radical with the two β-methylene protons having different dihedral angles with respect to the α2pπ spin-bearing orbital. Spin quantitation indicated that 13.2% of the *Da*SRE monomers had a radical. This improved radical formation compared to that of *Dg*ARE is likely due to better incorporation of [FeS] into *Da*SRE-AE (Figure S5FG).

Finally, we examined samples from assays containing either *Fp*TRE1 or *Fp*TRE2 and *Fp*TRE-AE. Formation of a radical species in either one of the TREs required NaDT, SAM, and TRE-AE. Both *Fp*TRE1 and *Fp*TRE2 show similar X-band EPR spectra of a protein-based carbon radical, with an intense feature at *g* = 2.004 and a low-field shoulder (Figure 2B).

The minor differences in the EPR spectra of the two TREs are likely attributable to subtle variations in the amino acid side chain orientation. Spin quantitation of the raw EPR spectra indicates 28% and 20% of *Fp*TRE1 and *Fp*TRE2 monomers, respectively, harbor a radical. Radical installation in a combined *Fp*TRE1 and *Fp*TRE2 sample appears to be simply the sum of the separate reactions.

The TRE EPR spectra are distinct from those of *Dg*ARE, *Da*SRE, and GREs, as they do not have resolved ^1^H hyperfine splittings (Figure 2B), and their assignment is not immediately obvious. As discussed in Table S6, Q-band (34.03 GHz) EPR spectra show that the TRE spectra are described by *g*-tensors with the minimal anisotropy of a carbon-centered radical, *g*_||_ = 2.004, *g*_⊥_ = 2.002 (Figure S7B). An oxygen-centered radical would have *g*_||_ ≈ 2.036, *g*_⊥_ ≈ 2.005,^65^ so the two *Fp*TRE EPR spectra cannot be assigned to an oxygen-centered radical (Figure 2A). Similarly, it could not be a β-carbon radical, for the remaining two methyl protons would give a quartet hyperfine pattern, as seen in the *Dg*ARE radical. Nor could it be a γ-carbon Thr radical, which is expected to have an EPR spectrum resembling the 5′-dA• radical, with ^1^H splittings from its two C–H protons and its one β-carbon proton.^66^ Moreover, homolytic bond cleavage of RH_2_C–H bonds (∼410 kJ mol^–1^)^67^ is on the higher end of that possible using 5′-dA• H-atom abstraction, making generation of this γ-carbon Thr radical species unlikely. The elimination of alternative forms of the Thr radicals leaves the α-carbon-centered radical as the best assignment. This assignment then implies that the hyperfine coupling to β-H is effectively nulled by a dihedral angle with respect to the α2pπ spin-bearing orbital of ∼90°.^63^

We performed additional paramagnetic resonance experiments to test this assignment of the TRE radical. X-band EPR spectra of *Fp*TRE1 and *Fp*TRE2 collected in D_2_O did not change the overall line shape compared to the H_2_O spectra (Figure S7C), indicating that the solvent-exchangeable ^1^H hyperfine coupling from N–H is small. This is corroborated in the Q-band ^1^H ENDOR (electron–nuclear double resonance) spectra of activated *Fp*TRE2 in H_2_O and D_2_O, which does not show signals from exchangeable ^1^H (Figure S7D). A possible explanation would involve a planar backbone conformation to minimize such coupling.^63^ Overall, these biochemical experiments reveal that AAREs are post-translationally modified by dedicated AARE-AEs that install previously unobserved α-carbon-centered radicals on Ala, Ser, and Thr (Figure 2C).

We cloned, expressed, and purified nine different point variants of the AAREs (Figure S8A). In these variants, the amino acid at the site of radical installation (*Dg*ARE A762, *Da*SRE S801, and *Fp*TRE1 T770) is substituted with either Gly, Ala, Ser, or Thr. To explore the importance of specific features of the Thr side chain for activation, we also constructed additional *Fp*TRE1 point variants by substituting Thr with Ile, Ser, or Cys. In addition, we constructed a variant of *Fp*TRE1 in which catalytic Cys437 is substituted with Ser. Analogous Cys variants of GREs are catalytically inactive but can still harbor a glycyl radical.^23,57,58,68,69^ Finally, we constructed C437S/T770G and C437S/T770A double variants of *Fp*TRE1.

**Figure 3:**
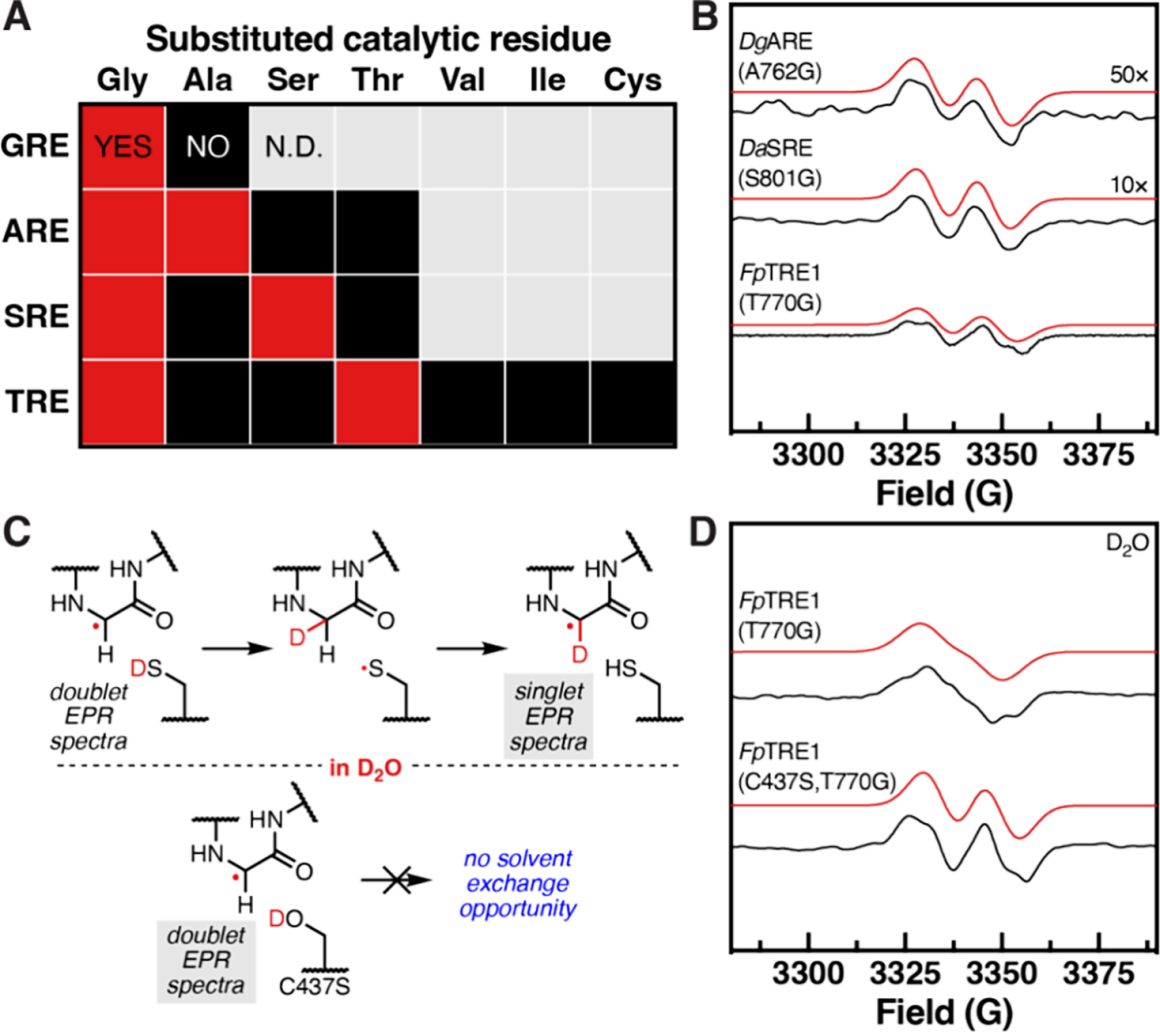
AARE-AEs have evolved to specifically recognize the different proteinogenic amino acids of AAREs. (A) Table of AARE point variants that can harbor a stable protein-based radical. Red boxes indicate radical formation; black, no radical formation; N.D., not determined. (B) Experimental (black) and simulated (red) CW X-band EPR spectra of AARE (X → G) point variants (77 K). Spectra are scaled to better distinguish features. (C) Model for solvent deuterium exchange of the glycyl radical via an intermediate thiyl radical. (D) Experimental (black) and simulated (red) CW X-band EPR spectra of *Fp*TRE1 (T770G) and *Fp*TRE1 (C437S/T770G) point variants in D_2_O (77 K).

We directly detected radicals generated on *Dg*ARE, *Da*SRE, and *Fp*TRE1 point variants by their respective activating enzymes by using X-band EPR spectroscopy. Notably, the only AARE point variants with detectable EPR signals were variants in which a Gly was introduced in place of Ala, Ser, or Thr to mimic the canonical GRE active site (Figure 3A and Figure S8BCD). In these cases, we observed the glycyl radical signal, although the percent radical installation was lower than in wild-type AARE in all cases, perhaps suggesting a lowered efficiency of activation or altered radical stability (Figure 3B). Activation of the Gly point variants could be a result of the conformational flexibility of Gly facilitating H-atom abstraction by the AARE-AEs. These results did not change with the *Fp*TRE1 double-point variants, as *Fp*TRE1 C437S/T770G formed a glycyl radical and *Fp*TRE1 C437S/T770A was not activated. For the *Fp*TRE1 variant, in which the catalytic Cys was substituted with Ser, the Thr radical was observed. These results highlight that although all AAREs contain l-amino acids with a similar α-carbon H-atom, the AARE-AEs appear to have evolved a preference for protein substrates containing specific amino acid side chains while retaining the ability to recognize the corresponding Gly-containing substrates.

Access to the *Fp*TRE1 C437S/T770G double variant also allowed us to indirectly assess whether the formation of the critical thiyl radical occurs in AAREs. The predicted bond distance between the catalytic residue and Cys of the AAREs (4.3–5.3 Å) is similar to those in X-ray crystal structures of GREs (3.7–4.9 Å). This key thiyl radical intermediate directly generates a substrate-based radical to initiate GRE catalysis (Figure 1B). In many GREs, activation in D_2_O results in a singlet rather than doublet EPR spectrum^32,58,70,71^ due to the nonstereoselective exchange of the Gly α-proton for a deuteron mediated by the catalytic Cys^72^ (Figure 3C). Consistent with past experiments, we observed collapse of the doublet in the EPR spectra of *Fp*TRE1 T770G prepared in D_2_O (Figure 3D). However, for the *Fp*TRE1 C437S/T770G variant lacking the putative active site Cys, we observed the canonical glycyl radical doublet, indicating no exchange. Altogether, these experiments are consistent with viable thiyl radical formation in AAREs and strongly suggest that these enzymes are catalytically active.

### Phylogenetic Analysis Suggests Broad Metabolic Roles for the AAREs

**Figure 4:**
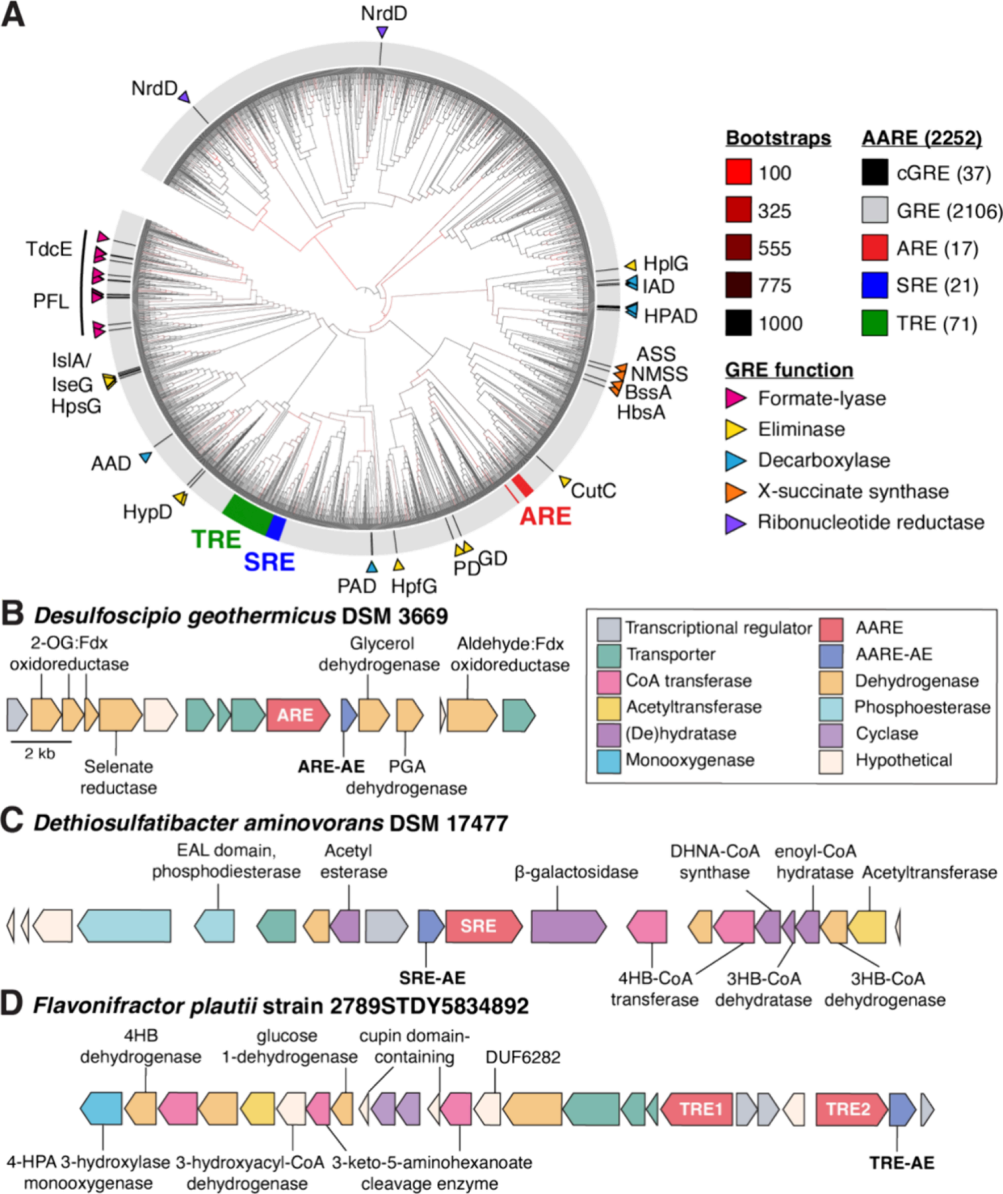
The AAREs likely evolved from GREs that catalyze elimination. (A) Maximum likelihood phylogenetic tree of the dereplicated nonredundant GRE superfamily (InterPro IPR004184). Edge colors correspond to bootstrap values. NrdD was used as the outgroup. Each leaf is labeled with the catalytic residue. Characterized GREs (cGRE) are indicated in black and labeled. Annotated genomic context for (B) the ARE-encoding cluster from *Desulfoscipio geothermicus* DSM 3669, (C) the SRE-encoding gene cluster from *Dethiosulfatibacter aminovorans* DSM 17477, and (D) two TRE-encoding gene clusters from *Flavonifractor plautii* strain 2789STDY5834892. Boundaries were chosen because they were the end of a contig or flanked by transcriptional regulators. Enzyme abbreviations are CutC (choline trimethylamine-lyase), HplG (*trans*-4-hydroxy-d-proline [d-Hyp] lyase), PD (1,2-propanediol dehydratase), HypD (*trans*-4-hydroxy-l-proline [l-Hyp] dehydratase), PFL (pyruvate formate-lyase), HpsG (2(*S*)-dihydroxypropanesulfonate [DHPS] sulfolyase), HpfG (DHPS-dehydratase), IslA/IseG (isethionate sulfite-lyase), HPAD (hydroxyphenylacetate decarboxylase), PAD (phenylacetate decarboxylase), IAD (indoleacetate decarboxylase), AAD (arylacetate decarboxylase), BssA (benzylsuccinate synthase), HbsA (hydroxybenzylsuccinate synthase), NMSS (naphthyl-2-methylsuccinate synthase), ASS (alkylsuccinate synthase), TdcE (ketobutyrate formate-lyase), and NrdD (ribonucleotide reductase).

Given that biochemical experiments suggest that the AAREs are functional enzymes, we next explored their evolutionary history to gain potential insights into their functions. In our phylogenetic tree, we find that each AARE clade forms a monophyletic group with robust bootstrap support, potentially suggesting that these Gly → (Ala, Ser, or Thr) mutations may have occurred only once in evolutionary history (Figure 4A,B). The AREs fall in a distinct clade from the SREs and TREs, perhaps indicating that they evolved separately. It is difficult to infer the evolutionary history of TREs from this analysis. Although TREs are located adjacent to the SREs (branching point bootstrap 800/1000), they are likely to be two isolated monophyletic groups that share a common ancestor in the tree.

We also explored the phylogenetic distribution of the AAREs, with the caveat that most sequences are derived from MAGs (Figure S9). The AAREs are widely distributed across diverse phyla, with the vast majority from the Eubacteriales order. Notably, the AREs tend to be found in *Candidatus* bacteria from extreme environments. The genomic contexts of the AAREs are varied, making the reaction prediction difficult. Attempts at hierarchical clustering of genomic neighborhoods^73^ did not reveal clear insights because very few classes of enzymes are shared across neighborhoods other than the rSAM enzyme superfamily (i.e., AARE-AEs) (Figure 4BCD). For the AARE sequences derived from MAGs, we cannot confidently determine whether these assemblies have captured the entire genomic context. However, in multiple instances, there is evidence that the neighboring regions encode additional enzymes, including those involved in anaerobic primary metabolic pathways. These data suggest that AAREs likely participate in multiple metabolic processes, and sampling more diverse environments may lead to the discovery of additional family members.

We predict that AAREs catalyze distinct reactions based on the low sequence identity they share with each other (17.3–99.5% amino acid identity [aa ID]) and with biochemically characterized GREs (14.5–47.3% aa ID). Most AAREs, including adjacently encoded TREs, share only 40–50% aa ID to one another, which is below the threshold that distinguishes most functionally distinct GREs.^23^ The AAREs share the highest percent amino acid identity to the GRE eliminases (21.1–47.3% aa ID), a subset of GREs that catalyze carbon–heteroatom bond cleavage reactions, suggesting that AAREs might catalyze related transformations. In support of this, the closest GRE clade to each AARE clade in the phylogenetic tree contains a biochemically characterized GRE eliminase (30–41/63–75% aa ID/similarity). The proposed substrate-binding pockets of the AAREs (Figure S10) are also most similar to those of structurally characterized GRE eliminases (Figure S11). Finally, to explore the biological relevance of the AAREs, we searched for AARE-encoding genes and transcripts in human microbiome metagenomics and metatranscriptomics data sets. Genes encoding AAREs are found at relatively high abundances comparable to those of genes encoding CutC^74^ and HPAD,^75^ gut microbial GREs that produce the disease-associated metabolites trimethylamine and *p*-cresol, respectively (Figure S12).

## Discussion

The GRE superfamily, which encompasses >25,000 predicted members (IPR004184), catalyzes a diverse range of reactions but was previously thought to universally require a conserved glycyl radical. We have discovered that a subset of these enzymes instead possesses a catalytic Ala, Ser, or Thr, greatly expanding the family. Each of these AAREs can be activated by its partner AARE-AE to install either an alanyl, serinyl, or threonyl radical, most likely via abstraction of the α-carbon H-atom. These three protein-centered radicals represent post-translational modifications that have not previously been observed in biochemistry. While these α-carbon radicals had been theorized to form adventitiously on proteins from exposure to reactive oxygen species,^43^ experimental studies indicate such radicals form exclusively on amino acid side chains rather than on α-carbon atoms.^76^ Generation of alanyl, serinyl, or threonyl radicals within short peptides typically requires X-ray irradiation or harsh reagents to produce these reactive intermediates.^77−80^ In our case, formation of these radical species occurs under neutral, mild, aqueous environments from the corresponding proteinogenic amino acids using dedicated post-translational modification enzymes.

Our unexpected observation of stable alanyl, serinyl, and threonyl radicals within the AAREs runs counter to the previous hypothesis that alanyl radicals do not occur in enzymes due to their predicted lower stability compared to glycyl radicals.^40,50^ The potential relevance of these radical intermediates was also suggested by past experiments with PFL-AE and the d-Ala-containing PFL peptide mimic, which indicated that alanyl radicals could form in a non-native context.^37^ Furthermore, recent work demonstrates the involvement of other aminoacyl Cα radicals on peptide substrates as catalytic intermediates.^20^ As computational studies have suggested, perhaps conformational flexibility of the glycyl radical, rather than radical stability, is the driving evolutionary pressure for the predominance of GREs in genomic data repositories.^40^ Notably, the wider set of radical intermediates we have revealed and characterized here are predicted to have thermodynamic stabilities similar to those of other protein-based radicals involved in enzyme catalysis (e.g., thiyl, tyrosyl, glycyl), a feature proposed to be important for radical enzyme function.^50^ These alternative protein-based radicals may have different vulnerabilities to O_2_, different impacts on protein stability, or altered catalytic rates due to differences in peptide backbone rigidity. Finally, results of our EPR experiments are consistent with the formation of a thiyl radical on the catalytic Cys of *Fp*TRE1, strongly suggesting that the AAREs are catalytically active.

The AARE-AEs most likely abstract H-atoms from the α-carbon of the nonglycyl catalytic residue, generating α-carbon radicals. While these C–H bonds are chemically similar to the C–H bond cleaved by the GRE-AEs, the α-carbon atoms of the catalytic residues in AAREs are chiral. This suggests that H-atom abstraction in AAREs occurs with a stereospecificity distinct from that proposed for GREs. It is unclear as to when and how this potential shift in stereospecificity arose evolutionarily and raises the question of whether all GRE-AEs abstract the *pro*-*S* H-atom of Gly, as has been universally accepted in the field.^37,56^ It is intriguing that the AARE-AEs exhibit such a limited scope, as all AAREs possess an l-amino acid with a similarly configured Cα–H bond. This narrow substrate scope suggests that AARE-AEs have evolved to recognize the side chains of specific amino acids, which further speaks to the biological relevance of these radical intermediates. Gly is the only residue identified thus far that can be activated when introduced into the AAREs, perhaps reflecting their ancestral origins. Moreover, AARE-AEs cannot cross activate AARE variants mutated to incorporate their preferred residue [*e.g*., TRE-AE cannot activate ARE(A → T) or SRE(S → T)], implying that additional factors mediate recognition (Figure S13).

To gain additional insight into the evolution and biological relevance of AAREs, it will be critical to identify specific reactions catalyzed by these enzymes, which are currently of unknown function. This is a major bottleneck in enzyme discovery more broadly and has been particularly challenging for AAREs due to the limited number of homologs present among bacterial isolates. Because the AAREs most closely resemble GRE eliminases, we hypothesize that they may mediate chemically analogous transformations. Future efforts will be focused on characterizing the metabolism of AARE-encoding organisms and uncovering the specific reactions catalyzed by the individual enzymes.

The discovery and characterization of new protein-based radicals greatly expand our understanding of radical enzymes. Prior to this work, the scope of protein-based radicals in biology was thought to be very constrained, relative to the number of proteinogenic amino acids. The discovery of alanyl, serinyl, and threonyl radicals nearly doubles the number of characterized protein-based radicals. While we have identified most AAREs in MAGs derived from the human microbiome, we are likely missing sequences from other anaerobic microbial habitats that are undersampled and undersequenced. AAREs have a relatively high occurrence in metagenomic repositories compared to many known GREs, and they have been identified in the genomes of diverse microbes. Although we have yet to link the AAREs to specific chemical reactions, our work suggests that they evolved to use these previously unanticipated protein-based radicals to mediate important functions. More broadly, the knowledge that these radical intermediates are viable within enzyme active sites enables their invocation in enzyme mechanisms outside of the AAREs. Altogether, this work not only lays the foundation for characterizing and investigating the expanded AARE protein family, thus challenging a paradigm of GRE biochemistry, but also suggests the existence of other previously unanticipated protein-based radical intermediates that await discovery.

## Supporting Information Available

Full description of materials, detailed procedures for plasmid construction, protein purification, *in vitro* biochemical assays, mass spectrometry analyses, phylogenetic analyses, Figures S1–S13, and Tables S1–S6 (PDF)
