# Supplementary material for: Discovery of a New Class of Aminoacyl Radical Enzymes Expands Nature’s Known Radical Chemistry
Source: J Am Chem Soc. 2024 Oct 11;146(43):29645–55. doi: 10.1021/jacs.4c10348 (PMC11528403; doi:10.1021/jacs.4c10348)
Supplement: Supplementary file 1 — ja4c10348_si_001.pdf [file ja4c10348_si_001.pdf]

Supporting Information

Discovery of a New Class of Aminoacyl Radical Enzymes Expands Nature's Known Radical Chemistry

Beverly Fu[1], Hao Yang[2], Duncan J. Kountz[1], Maike N. Lundahl[3], Harry R. Beller[4,5], William E. Broderick[3], Joan B. Broderick[3], Brian H. Hoffman[2], Emily P. Balskus*[1,6]

[1]Department of Chemistry and Chemical Biology, Harvard University, Cambridge, MA 02138, USA
[2]Department of Chemistry, Northwestern University, Evanston, IL 60208, USA
[3]Department of Chemistry and Biochemistry, Montana State University, Bozeman, MT 59717, USA
[4]Lawrence Berkeley National Laboratory, Berkeley, CA 94720, USA
[5]Department of Chemical Engineering and Applied Chemistry, University of Toronto, Toronto, ON M5S 3E5, Canada
[6]Howard Hughes Medical Institute, Harvard University, Cambridge, MA 02138, USA

Table of Contents

Supplemental figures S3
Figure S1. Non-glycyl radical radical enzymes (GREs) also employ glycyl radical intermediates S3
Figure S2. Multiple TREs are found adjacent to one TRE-AE S4
Figure S3. The AAREs are predicted to be structurally similar to the GREs S5
Figure S4. The AARE-AEs retain the [Fe_4S_4]-binding motifs. S6
Figure S5. AAREs and AARE-AEs are akin to GREs and GRE-AEs S8
Figure S6. *Fp*TRE-AE reductively cleaves SAM S10
Figure S7. The backbone amide proton of TREs does not couple to the *Fp*TRE1 radical S11
Figure S8. AARE-AEs are not promiscuous S12
Figure S9. AAREs are encoded by diverse microbes S14
Figure S10. Predicted substrate binding pockets of the AAREs S15
Figure S11. Substrate binding pockets of GREs S16
Figure S12. AAREs are found in human microbiome metagenomic samples S17
Figure S13. AARE-AEs cannot cross-activate other AAREs S18
Supplemental tables S19
Table S1. Accession numbers for identified alanyl radical enzymes (AREs) S19
Table S2. Accession numbers for identified serinyl radical enzymes (SREs) S20
Table S3. Accession numbers for identified threonyl radical enzymes (TREs) S21
Table S4. Strains and sequences used S23
Table S5. UPLC–MS/MS analysis of standards used for assays S28
Table S6. Hyperfine tensors (MHz) of simulated alanyl, serinyl, and threonyl radicals S29
General materials and methods S30
Bacterial strains S30
Plasmid construction S30
Expression and purification of heterologously expressed enzymes S31
Radical quantitation by electron paramagnetic resonance (EPR) spectroscopy S33
UV–vis assays of FeS cluster reduction S34
Generation of multiple sequence alignment and phylogenetic tree S34
Generation of predicted protein structures S34
UPLC–MS/MS assays for detecting end-point SAM cleavage products S34
Analysis of metagenomic and metatranscriptomic data S35
Supplemental references S36

Supplemental figures

Figure S1. Non-glycyl radical radical enzymes (GREs) also employ glycyl radical intermediates. (A) The radical S-adenosyl-L-methionine (rSAM) enzyme PhnJ generates a glycyl radical on its protein backbone, which abstracts a hydrogen atom (H-atom) from an adjacent Cys to form a thiyl radical and catalyze methane formation. (B) The Fe:Mn oxygenase enzyme CADD generates a backbone tyrosyl radical which can undergo side-chain cleavage to yield a glycyl radical and eventually *p*-aminobenzoate. (C) The diiron enzyme TglH excises the β-carbon of the terminal Cys of a short peptide. Following hydroxylation at the β-position, the substrate undergoes side-chain cleavage to form a glycyl radical, which can recombine with the Fe-bound thioformate. (D) The mononuclear non-heme Fe enzyme CalC reacts with the DNA-damaging diradical to form a backbone glycyl radical. (E) The copper and ascorbate-dependent monooxygenase PHM abstracts an H-atom from the terminal Gly of peptidylglycine to form a glycyl radical. Homolytic cleavage of the peroxide leads to the final product.

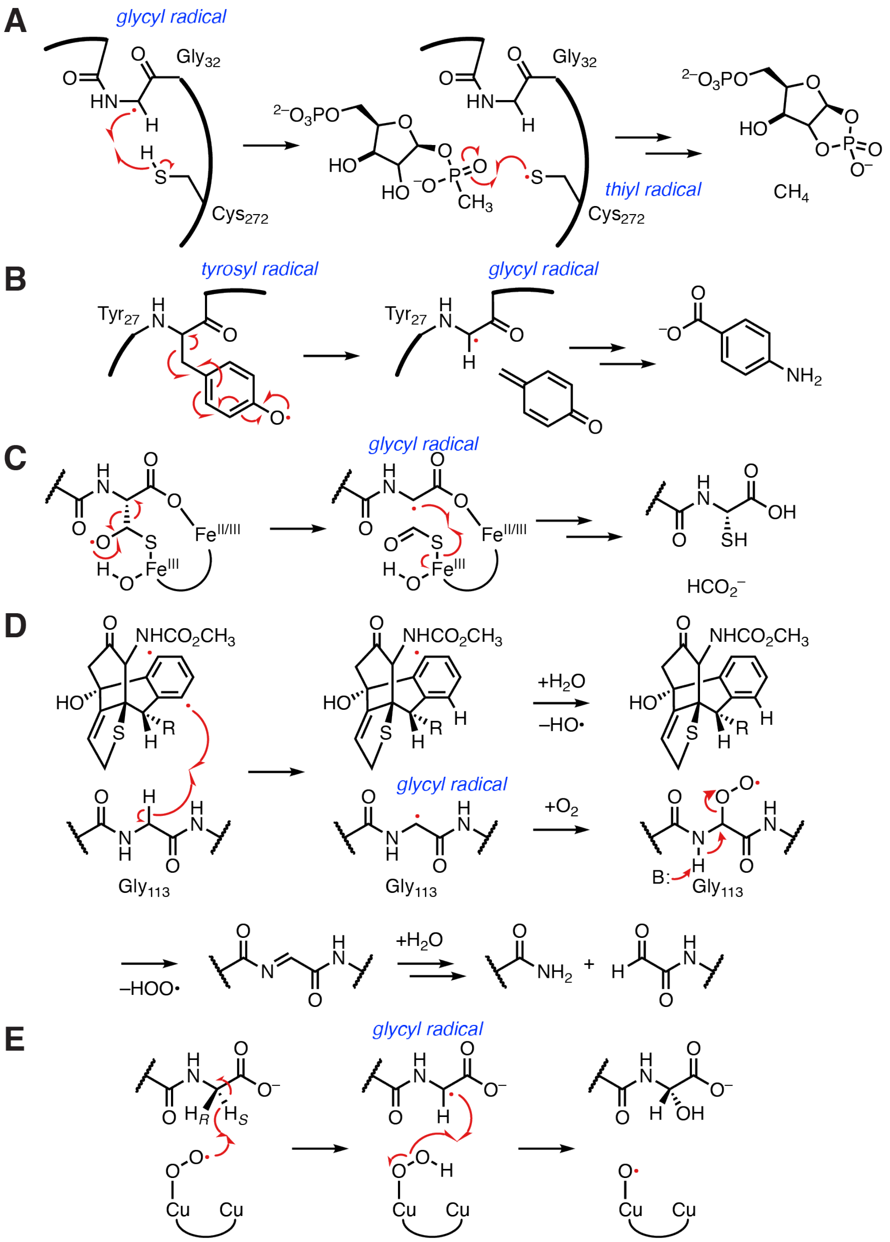

Figure S2. Multiple TREs are found adjacent to one TRE-AE. Gene clusters of TRE systems often include multiple TREs and a single TRE-AE.

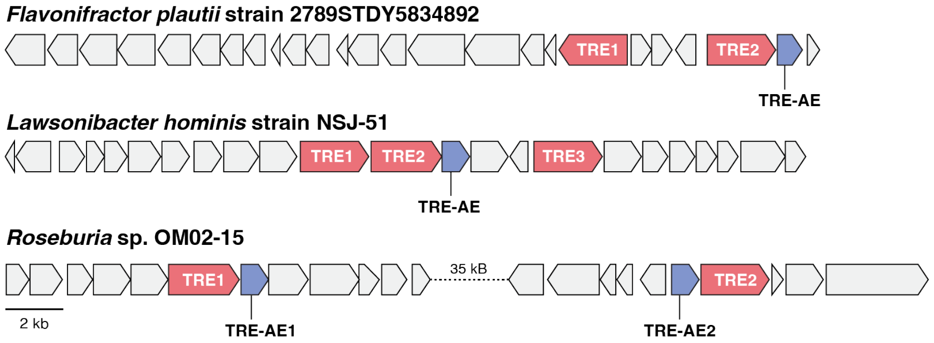

Figure S3. The AAREs are predicted to be structurally similar to the GREs. (A) The overall structural predictions of *Dg*ARE, *Da*SRE, *Fp*TRE1, *Fp*TRE2, and *Fp*TRE1–2 heterodimer retain the same secondary structure in comparison to the X-ray crystal structure of *E. coli* PFL (PDB accession code 2PFL). A predicted dimer interface is also conserved. Each monomer is colored white at the N-terminus and transitions to a colored C-terminus. (B) Key "Gly" and "Cys" structural loops and residues are similarly positioned among *Dg*ARE, *Da*SRE, *Fp*TRE1, *Fp*TRE2, and multiple GREs. Conserved catalytic residues are highlighted with the tan oval and bolded residue numbering.

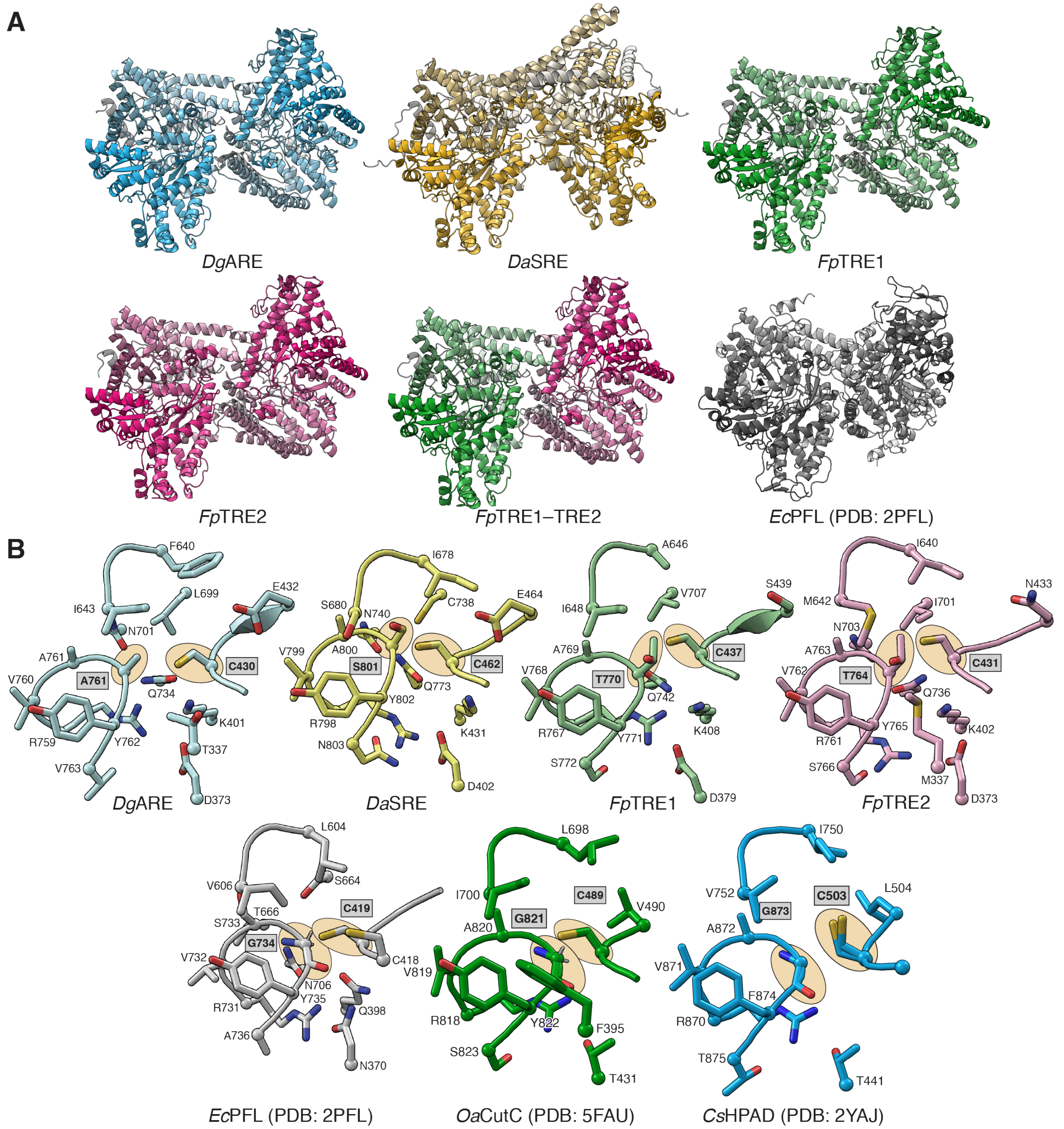

Figure S4. The AARE-AEs retain the [Fe_4S_4]-binding motifs. (A) Multiple sequence alignment (MSA) of the AARE-AEs against characterized GRE-AEs of the three [Fe_4S_4]-binding domains. (B) MSA of the SRE-AE homologs. We noticed that *Da*SRE-AE is missing one of the Cys (position 87) involved in ligating the second [Fe_4S_4]. Instead, a Thr is encoded and was confirmed by sequencing the genomic DNA of *D. aminovorans*. This appears to be the exception, as sequence alignments with the other SRE-AE homologs preserve the $CX_2CX_2CX_3$ motif. In addition, one other SRE-AE homolog from *Provencibacterium massiliense* Marseille-P2780 has a mutation within the first ferredoxin-binding motif (C56D). Cys predicted to bind the clusters are marked with the asterisk (*). (C) Structural predictions of *Dg*ARE-AE, *Da*SRE-AE, and *Fp*TRE-AE compared to the X-ray crystal structure of *Ec*PFL-AE (PDB accession code 3CB8) and the predicted structure of *Oa*CutD (Uniprot ID: Q30W71), which has additional auxiliary [FeS]. The SAM cofactor is in grey and the [Fe_4S_4] is depicted. Putative Cys involved in binding [FeS] are shown in red, stick format. (D) The amino acid percent sequence identity (top) and similarity (bottom) between the XRE-AEs, PFL-AE, and GRE-AEs of the eliminases are depicted.

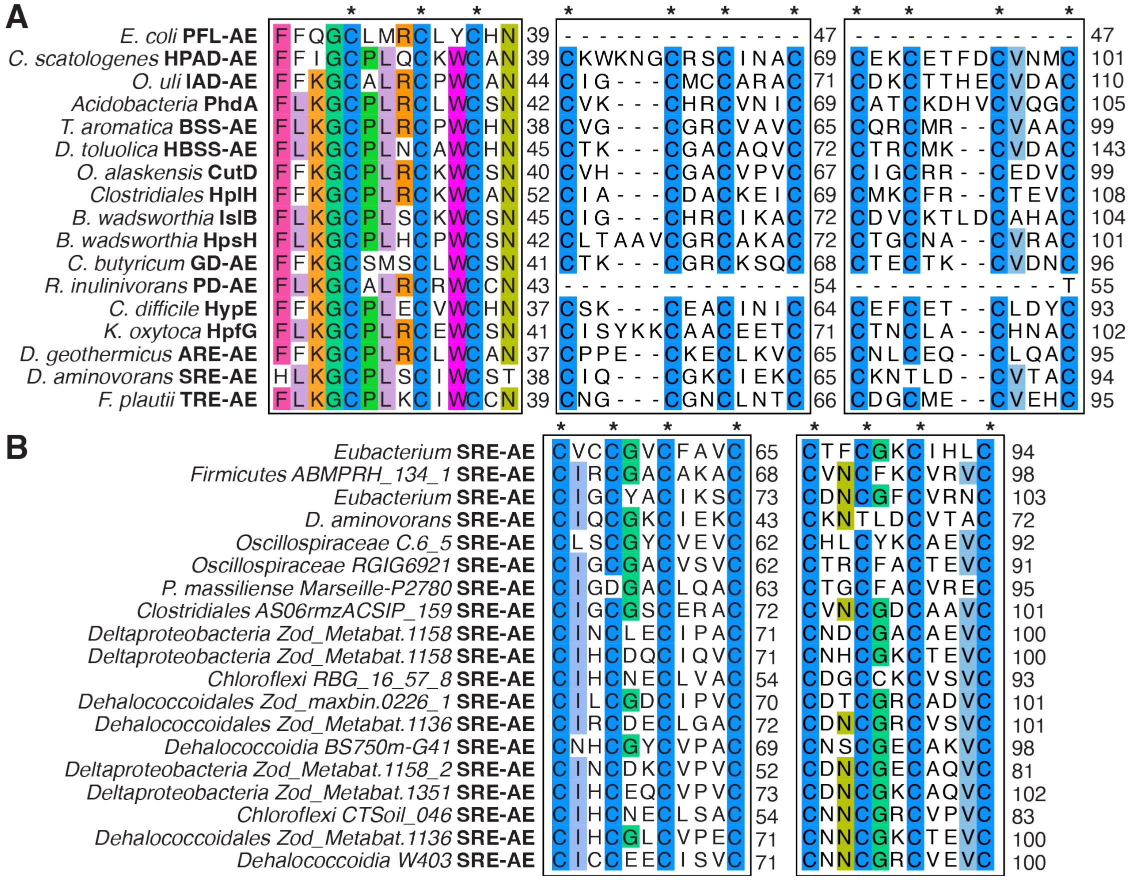

C

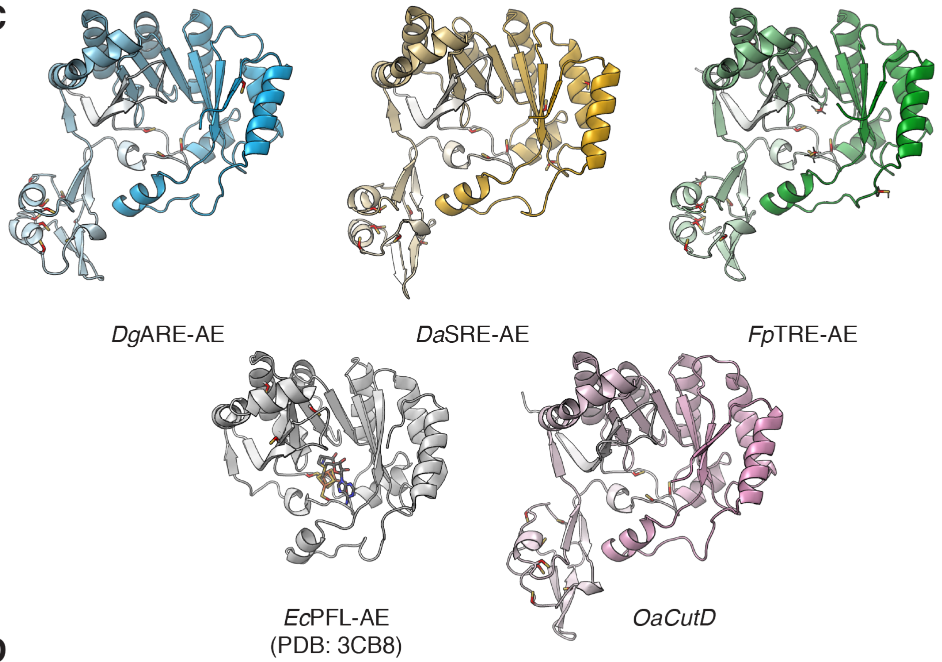

D

	PFL-AE	ARE-AE	SRE-AE	TRE-AE	HpfH	CutD	PD-AE	HypE	GD-AE	IslB	HpsH	HplH
P0A9N4 **PFL-AE**		28.5	20.9	22.2	26.0	25.0	30.8	25.7	29.6	25.4	23.1	28.8
D. geothermicus **ARE-AE**	28.5		33.0	38.1	33.4	38.4	29.9	39.8	35	38.2	36.1	38.8
D. aminovorans **SRE-AE**	20.9	33.0		32.5	33.0	32.6	28.6	32.7	36.1	35.8	32.2	35.3
F. plautii **TRE-AE**	22.2	38.1	32.5		32.1	34.2	32.9	38.9	35.1	31.7	36.5	37.3
A0A318FEA4 **HpfH**	26.0	33.4	33.0	32.1		32.2	31.3	33.5	38.1	33.5	33.2	38.4
Q30W71 **CutD**	25.0	38.4	32.6	34.2	32.2		31.2	39.5	36.9	38.6	39.7	37.0
ABC25540.1 **PD-AE**	30.8	29.9	28.6	32.9	31.3	31.2		29.2	34.8	29.8	32.1	32.4
A0A069AMK2 **HypE**	25.7	39.8	32.7	38.9	33.5	39.5	29.2		43.6	36.5	34.9	37.8
AAM54729.1 **GD-AE**	29.6	35.0	36.1	35.1	38.1	36.9	34.8	43.6		38.7	39.9	39.5
E5Y377 **IslB**	25.4	38.2	35.8	31.7	33.5	38.6	29.8	36.5	38.7		48.4	40.3
E5Y7I3 **HpsH**	23.1	36.1	32.2	36.5	33.2	39.7	32.1	34.9	39.9	48.4		41.9
A0A316Q2Y3 **HplH**	28.8	38.8	35.3	37.3	38.4	37	32.4	37.8	39.5	40.3	41.9	

	PFL-AE	ARE-AE	SRE-AE	TRE-AE	HpfH	CutD	PD-AE	HypE	GD-AE	IslB	HpsH	HplH
P0A9N4 **PFL-AE**		55.3	54.6	52.3	57.7	53.9	64.6	57.1	58.5	52.1	51.2	55.9
D. geothermicus **ARE-AE**	55.3		71.0	69.2	68.5	67.4	61.5	73.4	71.6	70.3	70.2	70.0
D. aminovorans **SRE-AE**	54.6	71.0		67.9	65.7	70.0	57.6	69.6	72.2	71.7	67.4	66
F. plautii **TRE-AE**	52.3	69.2	67.9		65.4	66.1	59.5	71.9	72.2	62.7	65.1	67.3
A0A318FEA4 **HpfH**	57.7	68.5	65.7	65.4		65.3	58.2	67.4	71.1	66.1	66.5	71.4
Q30W71 **CutD**	53.9	67.4	70.0	66.1	65.3		60.8	68.8	71.2	69.6	70.0	68.0
ABC25540.1 **PD-AE**	64.6	61.5	57.6	59.5	58.2	60.8		62.0	63.6	55.8	56.1	61.2
A0A069AMK2 **HypE**	57.1	73.4	69.6	71.9	67.4	68.8	62.0		75.6	66.4	65.4	72
AAM54729.1 **GD-AE**	58.5	71.6	72.2	72.2	71.1	71.2	63.6	75.6		69.4	69.3	71.8
E5Y377 **IslB**	52.1	70.3	71.7	62.7	66.1	69.6	55.8	66.4	69.4		76.5	68.9
E5Y7I3 **HpsH**	51.2	70.2	67.4	65.1	66.5	70.0	56.1	65.4	69.3	76.5		68.2
A0A316Q2Y3 **HplH**	55.9	70.00	66.0	67.3	71.4	68.0	61.2	72.0	71.8	68.9	68.2	

Figure S5. AAREs and AARE-AEs are akin to GREs and GRE-AEs. (A) SDS-PAGE gel of purified AAREs (2 µg per well). All proteins are His_6-tagged on the N-terminus. Precision Plus Protein All Blue Standards (Bio-Rad) (lane 1), *Dg*ARE-wildtype (WT) (lane 2), *Da*SRE-WT (lane 3), *Fp*TRE1-WT (lane 4), *Fp*TRE2-WT (lane 5), MBP–*Fp*TRE2 (lane 6). (B) SDS-PAGE gel of purified AARE-AEs (2 µg per well). All proteins are His_6-tagged on the N-terminus. Precision Plus Protein All Blue Standards (Bio-Rad) (lane 1), *Dg*ARE-AE (WT) (lane 2), *Da*SRE-AE (lane 3), *Fp*TRE1-AE (lane 4). (C) Size-exclusion chromatography (SEC) traces of *Dg*ARE, *Da*SRE, *Fp*TRE1, and *Fp*TRE2 compared to a standard. The molecular weights of the AARE monomers are ~90 kDa. Pulldown experiment of co-expressed *Fp*TRE1 and *Fp*TRE2 assessed by (D) SDS-PAGE and (E) α-Strep western blot. The arrow points to the expected molecular weight of the TREs (~90 kDa). Abbreviations are S (soluble fraction), F (flowthrough), and # (# mM imidazole). UV–vis absorption spectra of (F) *Dg*ARE-AE, (G) *Da*SRE-AE, and (H) *Fp*TRE-AE (± NaDT) (25 µM AARE-AE, 500 µM NaDT). Arrow points to the shoulder (~410 nm) that disappears upon reduction with sodium dithionite (NaDT), indicative of redox active [Fe_4S_4] clusters.

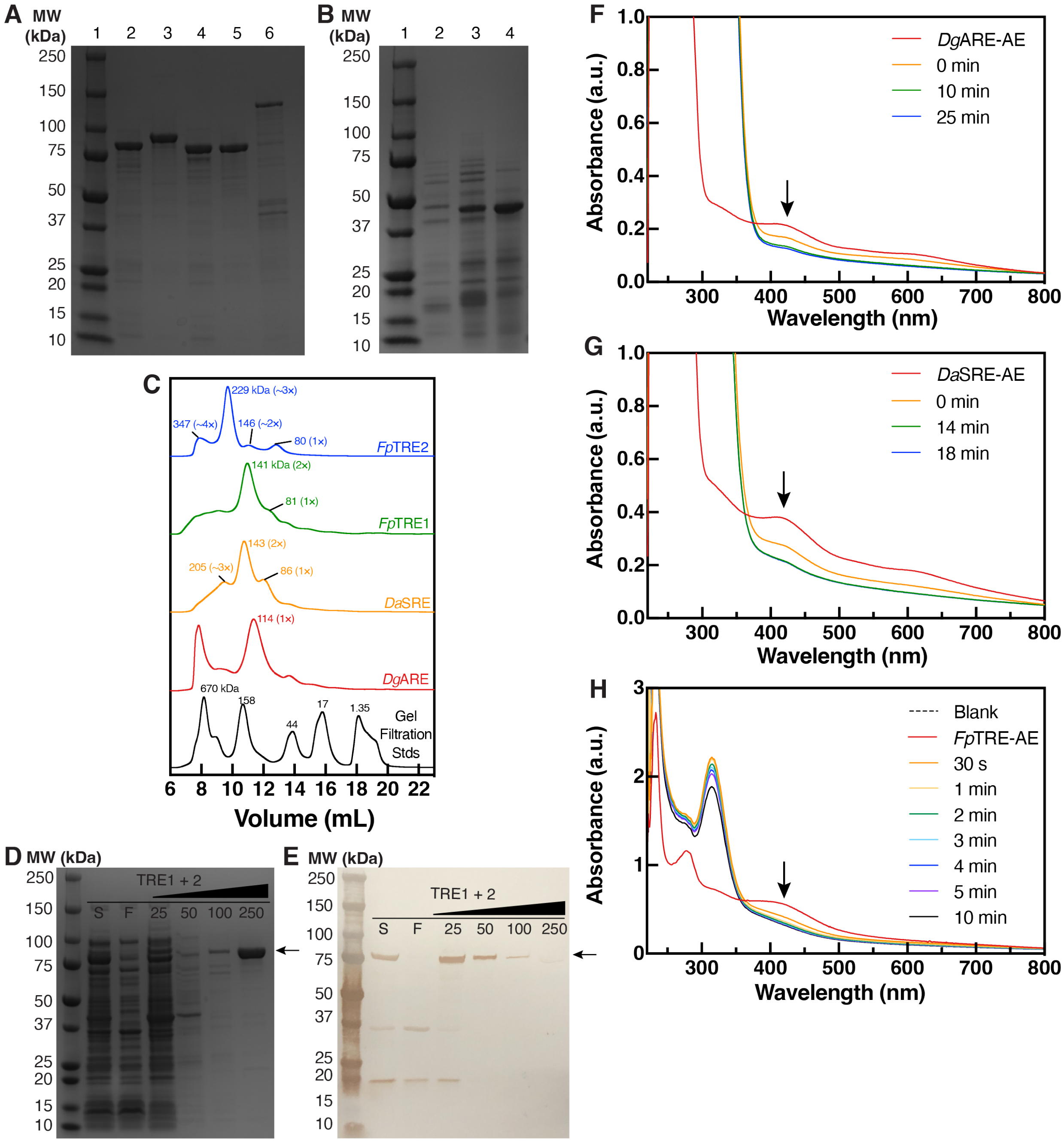

A MW (kDa) 1 2 3 4 5 6
250 150 100 75 50 37 25 20 15 10
B MW (kDa) 1 2 3 4
C
229 kDa (~3x)
347 (~4x)
146 (~2x)
80 (1x)
FpTRE2
141 kDa (2x)
81 (1x)
FpTRE1
143 (2x)
205 (~3x)
86 (1x)
DaSRE
114 (1x)
DgARE
670 kDa
158
44
17
1.35
Gel Filtration Stds
Volume (mL)
D MW (kDa)
TRE1 + 2
S F 25 50 100 250
E MW (kDa)
TRE1 + 2
S F 25 50 100 250
F
DgARE-AE
0 min
10 min
25 min
Absorbance (a.u.)
Wavelength (nm)
G
DaSRE-AE
0 min
14 min
18 min
Absorbance (a.u.)
Wavelength (nm)
H
Blank
FpTRE-AE
30 s
1 min
2 min
3 min
4 min
5 min
10 min
Absorbance (a.u.)
Wavelength (nm)

Figure S6. *Fp*TRE-AE reductively cleaves SAM. (A) CW X-band EPR spectra of reduced *Fp*TRE-AE without (black) and with (red) *S*-adenosyl-L-methionine (SAM) (12 K). (B) Reductive cleavage by *Fp*TRE-AE as shown by the time-dependent formation of 5′-dA. Formation of MTA is thought to arise from non-enzymatic processes. No SAH was observed. Measurements by UHPLC–MS/MS.

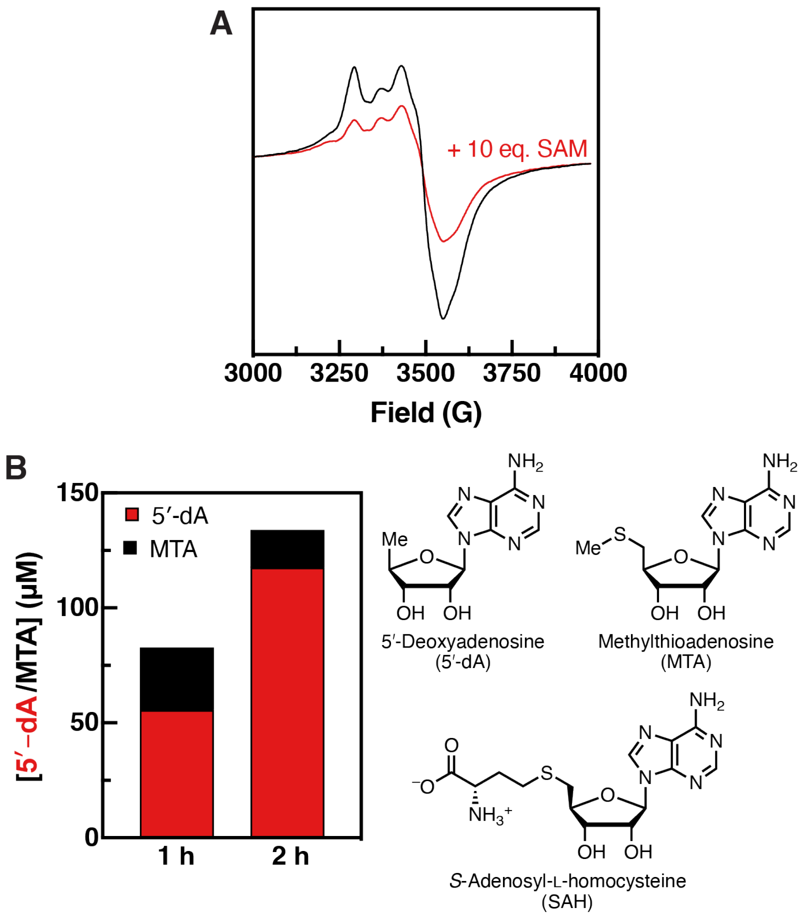

Figure S7. The backbone amide proton of TREs does not couple to the *Fp*TRE1 radical. (A) CW X-band EPR spectra of *Dg*ARE at various temperatures (4, 10, 77 K). (B) CW Q-band EPR spectra of *Fp*TRE1 and *Fp*TRE2 (40 K). (C) CW X-band EPR spectra of *Fp*TRE1 and *Fp*TRE2 in H_2O vs D_2O (40 K). (D) CW Q-band ^{1}H ENDOR spectra of *Fp*TRE2 in H_2O vs D_2O (2 K).

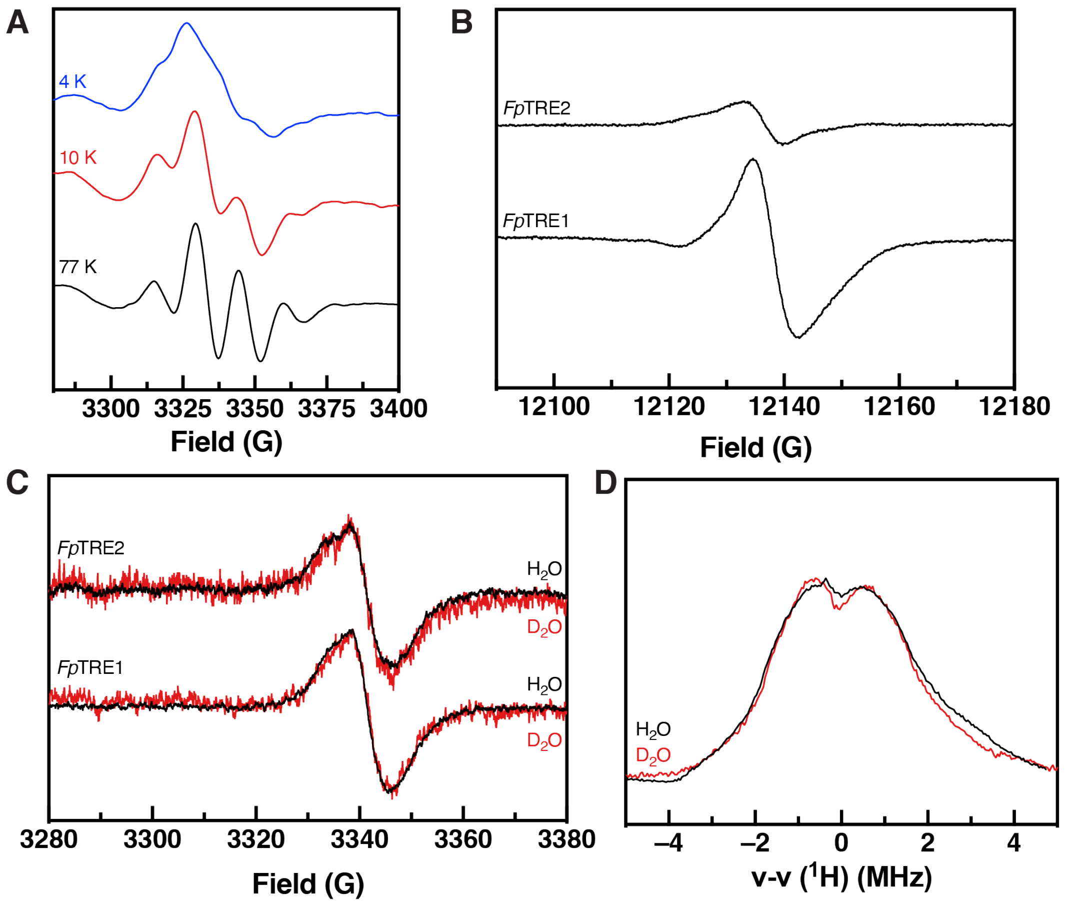

Figure S8. AARE-AEs are not promiscuous. (A) SDS-PAGE gel of purified AARE point variants (2 µg per well). All proteins are His_6-tagged on the N-terminus. Precision Plus Protein All Blue Standards (Bio-Rad) (lane 1), *Dg*ARE-A762G (lane 2), *Dg*ARE-A762T (lane 3), *Dg*ARE-A762S (lane 4), *Da*SRE-S801G (lane 5), *Da*SRE-S801T (lane 6), *Da*SRE-S801A (lane 7), *Fp*TRE1-T770G (lane 8), *Fp*TRE1-T770A (lane 9), *Fp*TRE1-C437S (lane 10), *Fp*TRE1-C437S/T770G (lane 11), *Fp*TRE1-C437S/T770A (lane 12), *Fp*TRE1-T770V (lane 13), *Fp*TRE1-T770I (lane 14), *Fp*TRE1-T770S (lane 15), *Fp*TRE1-T770C (lane 16). CW X-band EPR spectra of (B) *Dg*ARE, (C) *Da*SRE, and (D) *Fp*TRE1 point variants (77 K).

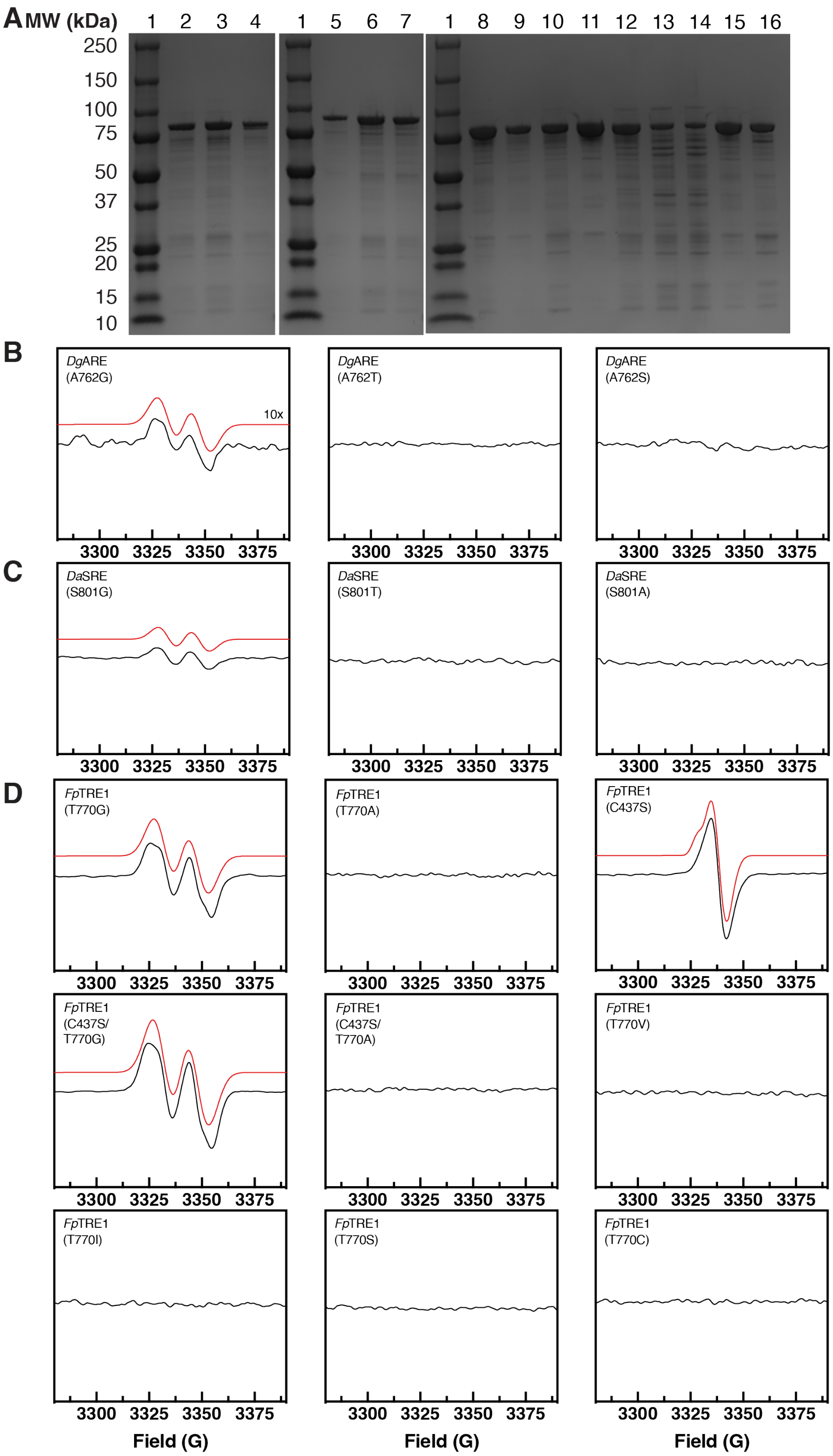
A
MW (kDa)
1 2 3 4 1 5 6 7 1 8 9 10 11 12 13 14 15 16
250
150
100
75
50
37
25
20
15
10
B
DgARE (A762G)
10x
DgARE (A762T)
DgARE (A762S)
C
DaSRE (S801G)
DaSRE (S801T)
DaSRE (S801A)
D
FpTRE1 (T770G)
FpTRE1 (T770A)
FpTRE1 (C437S)
FpTRE1 (C437S/ T770G)
FpTRE1 (C437S/ T770A)
FpTRE1 (T770V)
FpTRE1 (T770I)
FpTRE1 (T770S)
FpTRE1 (T770C)
3300 3325 3350 3375
Field (G)

Figure S9. AAREs are encoded by diverse microbes. (A) ARE, (B) SRE, and (C) TRE clades of AARE/GRE phylogenetic tree (amino acid sequence) presented in **Figure 4A**. The predicted encoding microbial species is annotated when available.

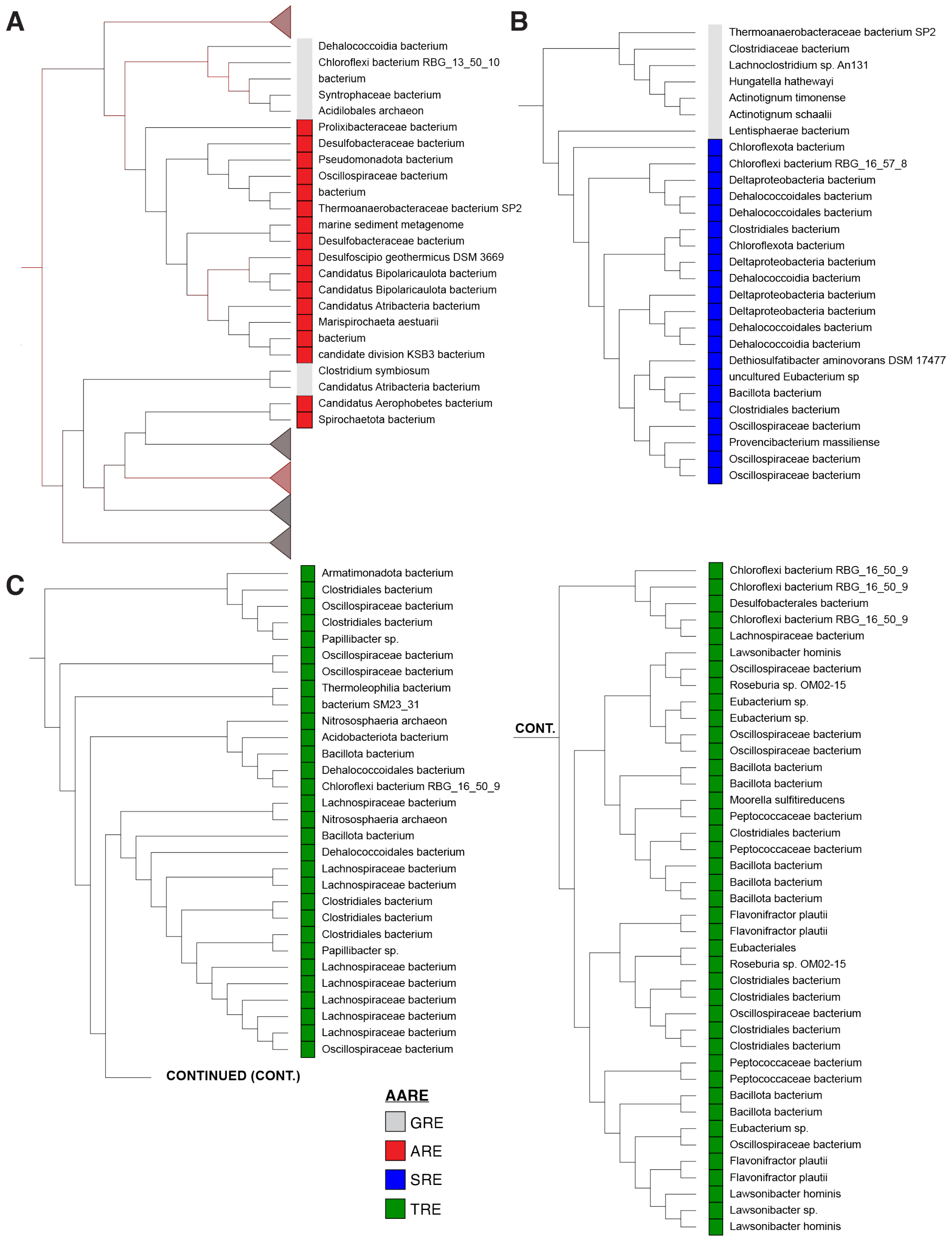

Figure S10. Predicted substrate binding pockets of the AAREs. Predicted active site of *Dg*ARE, *Da*SRE, *Fp*TRE1, and *Fp*TRE2 were determined by overlaying with the active sites of GRE X-ray crystal structures. Residues 4 Å away from the presumed substrate binding pocket are depicted and labeled.

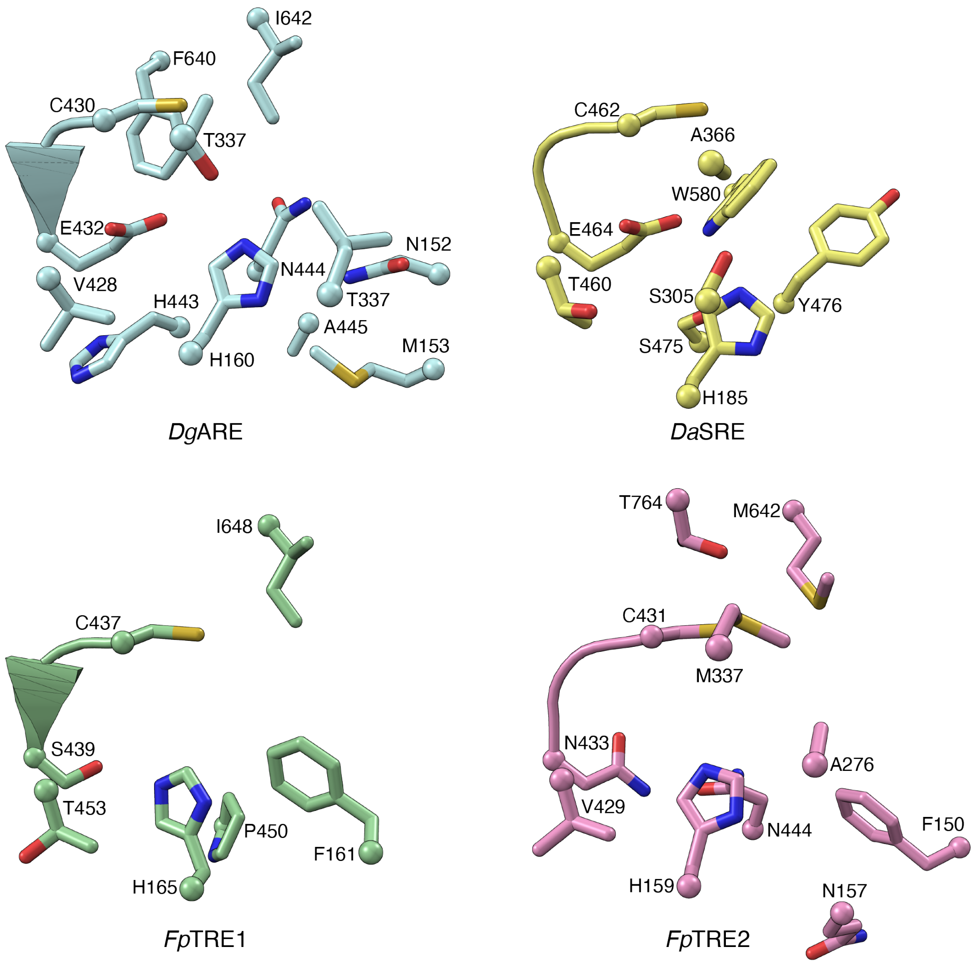

Figure S11. Substrate binding pockets of GREs. Residues within 4 Å of the bound ligands (white) are depicted, as well as proposed key interactions critical for substrate binding. PFL + pyruvate (PDB accession code 1H16), GD + glycerol (PDB accession code 1R9D), PD + (*S*)-1,2-propanediol (PDB accession code 5I2G), HypD + 4-hydroxy-L-proline (PDB accession code 6VXE), HplG + 4-hydroxy-D-proline (PDB accession code 7VUA), CutC + choline (PDB accession code 5FAU), HpsG + (*S*)-2,3-dihydroxypropane-1-sulfonate (PDB accession code 6LON), IslA + isethionate (PDB accession code 7KQ3), BSS + toluene + fumarate (PDB accession code 5BWE), and HPAD + *p*-hydroxyphenylacetate (PDB accession code 2YAJ). Residues that form interactions with the substrate are labeled.

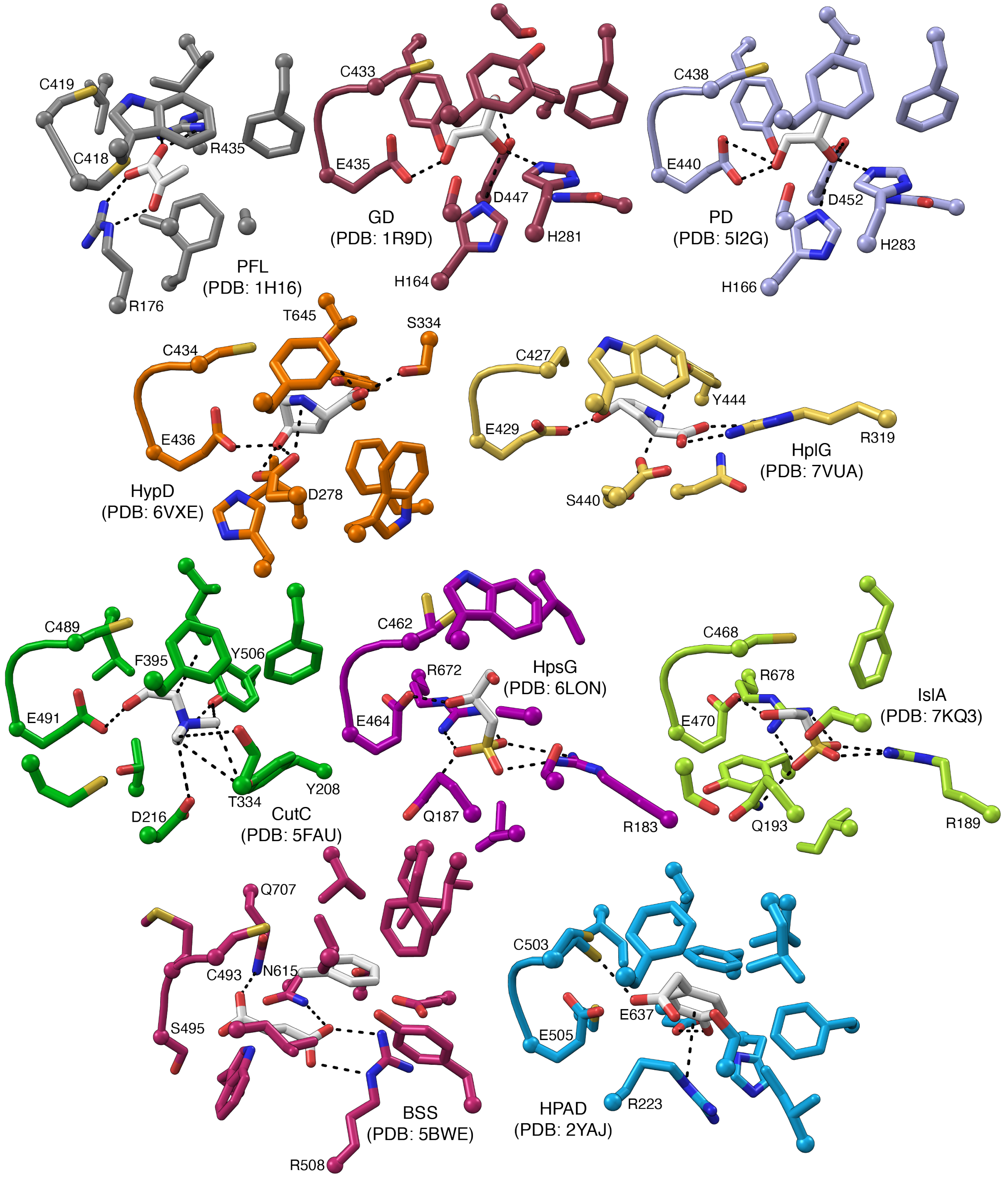

Figure S12. AAREs are found in human microbiome metagenomic samples. Metagenomic and metatranscriptomic data analyses of GRE (PFL, HPAD, CutC; 62% amino acid ID cutoff) and AAREs (ARE, TRE, SRE; 90% aa ID) from (A) Men's Lifestyle Validation Study (MLVS) PRJNA354235[1] (913 metagenomics [MGX], 378 metatranscriptomics [MTX] samples) (B) HMP2 PRJNA398089[2] (1338 MGX, 762 MTX), and (C) iHMP datasets PRJNA389280[3] (300 MGX, 78 MTX).

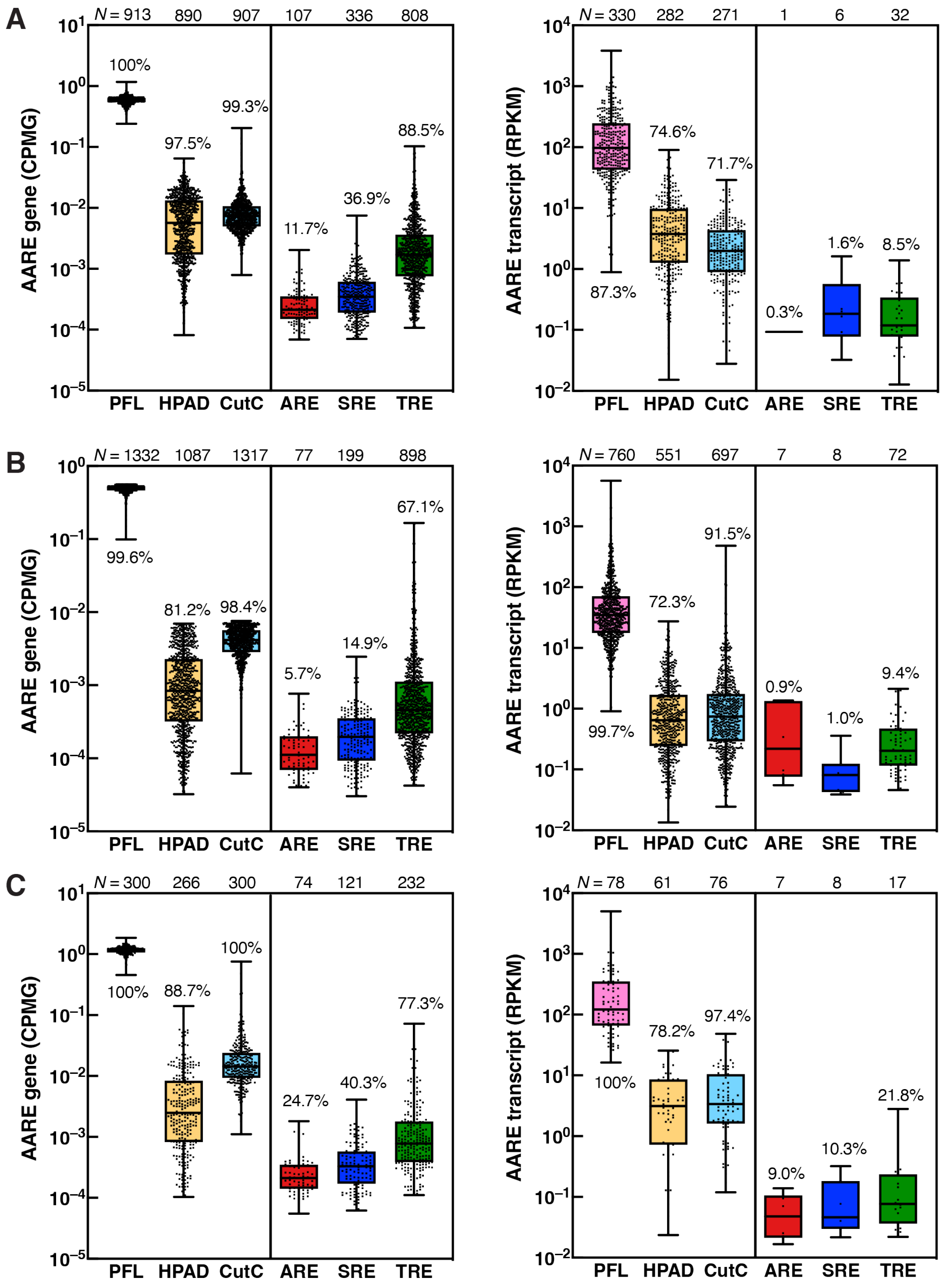

Figure S13. AARE-AEs cannot cross-activate other AAREs. X-band EPR spectra of cross-reacting X_1RE-AE/X_2RE($X_2 \rightarrow X_1$) pairs (77 K).

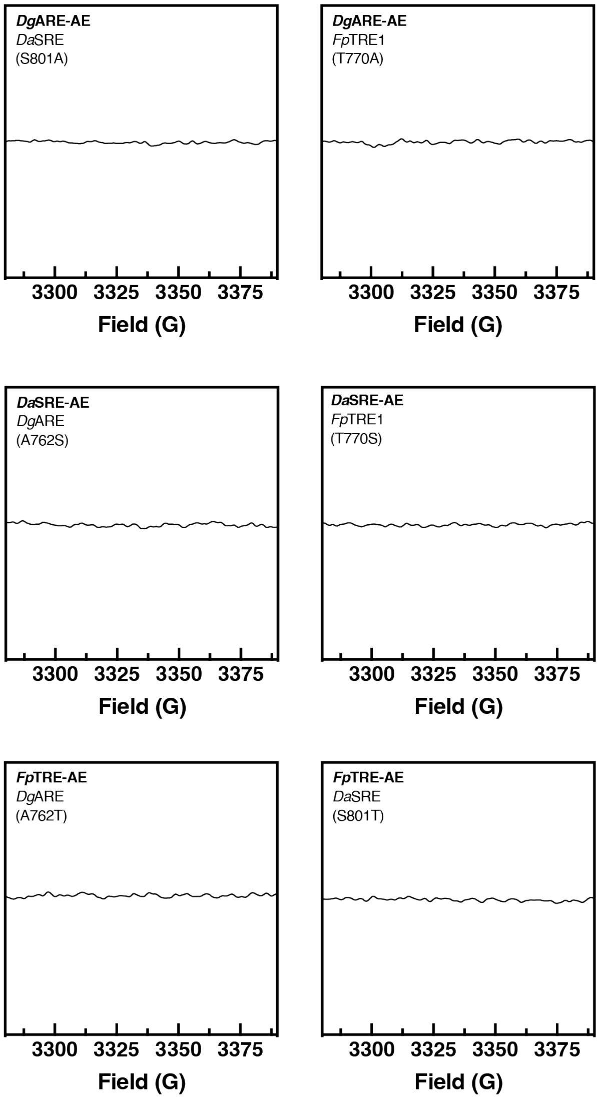

Supplemental tables

Table S1. Accession numbers for identified alanyl radical enzymes (AREs). Sequences from metagenome assembled genomes (MAGs) are indicated.

Uniprot/Uniparc	NCBI	MAG?	Organism
	MCP4405061	x	bacterium isolate Cca_15
	MCK4824285	x	bacterium isolate RS_11_71
UPI0019F95FFD	MBD3306905	x	Candidate division KSB3 isolate Bin_567
	MCD6317640	x	Candidatus Aerophobetes isolate AUK284
A0A7C0W2D6	HDK27323	x	Candidatus Atribacteria isolate HyVt-22
	MCK4392457	x	Candidatus Bipolaricaulota bacterium isolate RS_16_26
	MCK4600350	x	Candidatus Bipolaricaulota isolate RS_12_53
	MCK4785092	x	Desulfobacteraceae isolate RS_11_58
	MCK4793749	x	Desulfobacteraceae isolate RS_11_58
A0A1I6D5X9	WP_092482328		Desulfoscipio geothermicus DSM 3669
X1FH99	GAH45001	x	marine sediment metagenome
A0A1Y1S1V7	WP_083047660		Marispirochaeta aestuarii
	MBQ3276409	x	Oscillospiraceae isolate RGIG2395
A0A350YYY1	HAX96749	x	Prolixibacteraceae isolate UBA8917
	MBS1214180	x	Proteobacteria bacterium isolate CTSoil_133
	MBN2322287	x	Spirochaetes isolate Zod_Metabat.958
A0A3A9K1M6	RKL64051		Thermoanaerobacteraceae SP2

Table S2. Accession numbers for identified serinyl radical enzymes (SREs). Sequences from metagenome assembled genomes (MAGs) are indicated.

Uniprot/Uniparc	***NCBI***	***MAG?***	***Organism***
	MBP1706870	x	*Chloroflexi* bacterium isolate CTSoil_046
A0A1F8QZG3	OGO40451	x	Chloroflexi bacterium RBG_16_57_8
	MCL5959289	x	*Chloroflexi* isolate FK_Sedi_B_Bin.187
A0A6N9QAX2		x	*Clostridiales* bacterium
UPI00169A31D2	NLT40659	x	*Clostridiales* isolate AS06rmzACSIP_159
	MBN2239503	x	*Dehalococcoidales* bacterium isolate Zod_maxbin.0226_1
	MBN2074660	x	*Dehalococcoidales* bacterium isolate Zod_Metabat.1136
	MBN2077277	x	*Dehalococcoidales* bacterium isolate Zod_Metabat.1136
A0A7C3C016	HEY95974	x	*Dehalococcoidia* bacterium isolate W403
	MBL7120311	x	*Dehalococcoidia* isolate BS750m-G41
	MBN2060038	x	*Deltaproteobacteria* bacterium isolate Zod_Metabat.1158
	MBN2060064	x	*Deltaproteobacteria* isolate Zod_Metabat.1158
	MBN2062047	x	*Deltaproteobacteria* isolate Zod_Metabat.1158
UPI001ECBAB71	MBN1831961	x	*Deltaproteobacteria* isolate Zod_Metabat.1351
A0A1M6EMU2	WP_073048690		*Dethiosulfatibacter aminovorans* DSM 17477
	MCI9595688	x	*Firmicutes* isolate ABMPRH_134_1
	MCD8100426	x	*Oscillospiraceae* bacterium isolate C.6_5
	MBR6206454	x	*Oscillospiraceae* isolate RGIG6921
	MCR5173060	x	*Oscillospiraceae* isolate RUI082
A0A3E2T8H1	WP_079699170		*Provencibacterium massiliense* Marseille-P2780
A0A1C6H9F0	SCJ54209	x	uncultured *Eubacterium* sp.

Table S3. Accession numbers for identified threonyl radical enzymes (TREs). Sequences from metagenome assembled genomes (MAGs) are indicated. Gene ID JGI2065J20421_10059772 from Genome ID 3300001865 in the JGI IMG/MER database was a TRE initially identified in a sewage-derived metagenome.[4]

Uniprot/Uniparc	*NCBI*	*MAG?*	*Organism*
UPI001B45AA92	MBP1608018	x	*Acidobacteria* bacterium isolate CTSoil_011
	MBN1461875	x	*Armatimonadetes* bacterium isolate Zod_Metabat.349
A0A0S8IIS4	KPK96364	x	bacterium SM23_31 isolate SM23_31
A0A1F8PCN9	OGO20611	x	*Chloroflexi* bacterium RBG_16_50_9
A0A1F8PH92	OGO22533	x	*Chloroflexi* bacterium RBG_16_50_9
A0A1F8PP25	OGO24933	x	*Chloroflexi* bacterium RBG_16_50_9
A0A1F8PQB3	OGO25384	x	*Chloroflexi* bacterium RBG_16_50_9
UPI001691FF9D	NLT39461	x	*Clostridiales* bacterium isolate AS06rmzACSIP_159
UPI0016A1F266	NLV49852	x	*Clostridiales* bacterium isolate AS06rmzACSIP_36
UPI00168E9DD5	NLV50172	x	*Clostridiales* bacterium isolate AS06rmzACSIP_36
UPI00169454BB	NLM84989	x	*Clostridiales* bacterium isolate AS20ysBPTH_149
UPI00169F43BA	NLM84711	x	*Clostridiales* bacterium isolate AS20ysBPTH_149
	MCL2493377	x	*Clostridiales* bacterium isolate Lab288P4bin39
	MCL2493521	x	*Clostridiales* bacterium isolate Lab288P4bin39
	MCL1895360	x	*Clostridiales* bacterium isolate Nt197P3bin136
	MCL1895359	x	*Clostridiales* bacterium isolate Nt197P3bin136
	NTV91016	x	*Clostridiales* bacterium isolate W4_Combined_metabat1_078
	MBN1367781	x	*Dehalococcoidales* bacterium isolate Zod_Metabat.409
	MBN1191592	x	*Dehalococcoidales* bacterium isolate Zod_Metabat.59
	MCJ7785099	x	*Desulfobacterales* bacterium isolate PFL119
	WP_118702749	x	*Eubacterium ramulus* isolate MGYG-HGUT-02278
	MCR5784199	x	*Eubacterium* sp. isolate RUI358
	MCR5784486	x	*Eubacterium* sp. isolate RUI358
	MCR4842003	x	*Eubacterium* sp. isolate RUI539
UPI0016A4B17C	NLY38886	x	*Firmicutes* bacterium isolate AS09scLD_241
UPI0016A26AB8	NLY39532	x	*Firmicutes* bacterium isolate AS09scLD_241
	MBP2643104	x	*Firmicutes* bacterium isolate CTSoil_074
	MBP2643915	x	*Firmicutes* bacterium isolate CTSoil_074
UPI001D7F8C64	MBP2652800	x	*Firmicutes* bacterium isolate CTSoil_080
	MBP2650375	x	*Firmicutes* bacterium isolate CTSoil_081
	MBP2651948	x	*Firmicutes* bacterium isolate CTSoil_081
	MBP2649187	x	*Firmicutes* bacterium isolate CTSoil_081
	MBP2645149	x	*Firmicutes* bacterium isolate CTSoil_082
UPI0005D20086	WP_044945557		*Flavonifractor plautii* strain DFI.2.29
UPI001FAE1482	WP_243280625		*Flavonifractor plautii* strain DFI.2.29
H1CLJ0	WP_009261133		*Flavonifractor plautii* strain 2789STDY5834892
H1CLJ4	WP_009261137		*Flavonifractor plautii* strain 2789STDY5834892
	MBQ1285660	x	*Lachnospiraceae* bacterium isolate RGIG477
	MBR2755104	x	*Lachnospiraceae* bacterium isolate RGIG5612
	MBR3262066	x	*Lachnospiraceae* bacterium isolate RGIG5841
	MBR3362249	x	*Lachnospiraceae* bacterium isolate RGIG5887
	MBR3396516	x	*Lachnospiraceae* bacterium isolate RGIG5918
	MBR3396626	x	*Lachnospiraceae* bacterium isolate RGIG5918
	MBR3396618	x	*Lachnospiraceae* bacterium isolate RGIG5918
	MBR5930648	x	*Lachnospiraceae* bacterium isolate RGIG7843
	MCD7819850	x	*Lachnospiraceae* bacterium isolate U.3_11

A0A8J6J4A9	WP_186906260		*Lawsonibacter hominis* strain NSJ-51
A0A8J6M7K3	WP_186906265		*Lawsonibacter hominis* strain NSJ-51
A0A8J6M8C9	WP_186906264		*Lawsonibacter hominis* strain NSJ-51
	MCI6399270	x	*Lawsonibacter* sp. isolate SUG668 k141_68270
	WP_258359133		*Moorella* sp. SLA38
A0A7J3XKJ4			*Nitrososphaeria archaeon*
A0A7J3XLP1			*Nitrososphaeria archaeon*
	MCI8539015	x	*Oscillospiraceae* bacterium isolate ABMPRH_156_1
	MCD7846338	x	*Oscillospiraceae* bacterium isolate MO.B6_13
	MBR1779941	x	*Oscillospiraceae* bacterium isolate RGIG4999
	MBR1780173	x	*Oscillospiraceae* bacterium isolate RGIG4999
	MBR1781143	x	*Oscillospiraceae* bacterium isolate RGIG4999
	MBR4157526	x	*Oscillospiraceae* bacterium isolate RGIG6955
	MBR4157575	x	*Oscillospiraceae* bacterium isolate RGIG6955
UPI001B206140	MBO7729163	x	*Oscillospiraceae* bacterium isolate RGIG9615
	MCC8121923	x	*Oscillospiraceae* bacterium isolate U_126913
A0A7C6FVH9	HHT17664	x	*Papillibacter* sp. isolate AS08sgBPME_352
A0A7C6FVN0	HHT17011	x	*Papillibacter* sp. isolate AS08sgBPME_352
	MCR4443345	x	*Peptococcaceae* bacterium isolate A05DMB06
	MCR4443005	x	*Peptococcaceae* bacterium isolate A05DMB06
	MCR4443009	x	*Peptococcaceae* bacterium isolate A05DMB06
	MCL6477317	x	*Peptococcaceae* bacterium isolate HOT.MB2.86
A0A3R6XY18			*Roseburia* sp. OM02-15
A0A3R6UFF6	WP_118702726		*Roseburia* sp. OM02-15
	MBN1321014	x	*Thermoleophilia* bacterium isolate Zod_Metabat.495

Table S4. Strains and sequences used. Strains (A), plasmids (B), oligonucleotides (C), and codon optimized nucleotide sequences (D) used.

A. *E. coli* strains

Strain	*Genotype*	*Source*
Top10	F^{-}*mcr*A Δ(*mrr-hsd*RMS-*mcr*BC) ϕ80*lac*ZΔM15 Δ*lac*X74 *rec*A1 *ara*D139 Δ(*ara-leu*)7697 *gal*U *gal*K λ^{-} *rps*L(Str^{R}) *end*A1 *nup*G	Invitrogen
BL21(DE3)	F^{-}*omp*T *hsd*S_B (r_B^{-}, m_B^{-}) *gal dcm* (DE3)	Invitrogen
BL21(DE3) ΔiscR	F^{-}*omp*T *hsd*S_B (r_B^{-}, m_B^{-}) *gal dcm* (DE3) Δ*iscR*	Levin et al.[5]
BL21(DE3), CodonPlus-RIL	F^{-}*omp*T *hsd*S (r_B^{-}, m_B^{-}) *dcm*$^{+}$ Tet^{r} *gal* λ(DE3) *endA* Ht*e* [*argU ileY leuW* Cam^{r}]	Agilent
BL21(DE3) ΔiscR, CodonPlus-RIL	F^{-}*omp*T *hsd*S (r_B^{-}, m_B^{-}) *dcm*$^{+}$ Tet^{r} *gal* λ(DE3) *endA* Ht*e* [*argU ileY leuW* Cam^{r}] Δ*iscR*	Levin et al.[5]

B. Plasmids

Plasmid	*Description*	*Source*
pET28a-FpTRE1	*His_6-FpTRE1 (T7), lacI, Km^r, pBR322*	This study
pET28a-FpTRE2	*His_6-FpTRE2 (T7), lacI, Km^r, pBR322*	This study
pET-FpTRE-AE	*His_6-SUMO-FpTRE-AE (T7), lacI, Amp^r, pBR322*	This study
pET28a-FpTRE1(T770G)	*His_6-FpTRE1(T770G) (T7), lacI, Km^r, pBR322*	This study
pET28a-FpTRE1(T770A)	*His_6-FpTRE1(T770A) (T7), lacI, Km^r, pBR322*	This study
pET28a-FpTRE1(C437S)	*His_6-FpTRE1(C437S) (T7), lacI, Km^r, pBR322*	This study
pET28a-FpTRE1(C437S/T770G)	*His_6-FpTRE1(C437S/T770G) (T7), lacI, Km^r, pBR322*	This study
pET28a-FpTRE1(C437S/T770A)	*His_6-FpTRE1(C437S/T770A) (T7), lacI, Km^r, pBR322*	This study
pET28a-FpTRE1(T770V)	*His_6-FpTRE1(T770V) (T7), lacI, Km^r, pBR322*	This study
pET28a-FpTRE1(T770I)	*His_6-FpTRE1(T770I) (T7), lacI, Km^r, pBR322*	This study
pET28a-FpTRE1(T770S)	*His_6-FpTRE1(T770S) (T7), lacI, Km^r, pBR322*	This study
pET28a-FpTRE1(T770C)	*His_6-FpTRE1(T770C) (T7), lacI, Km^r, pBR322*	This study
pPR-IBA2-FpTRE2	*Strep-FpTRE2 (T7), lacI, Amp^r, f1*	This study
pET28a-DgARE	*His_6-DgARE (T7), lacI, Km^r, pBR322*	Twist Biosciences
pET28a-DgARE-AE	*His_6-SUMO-DgARE-AE (T7), lacI, Km^r, pBR322*	Twist Biosciences
pET28a-DgARE(A762G)	*His_6-DgARE(A762G) (T7), lacI, Km^r, pBR322*	This study
pET28a-DgARE(A762T)	*His_6-DgARE(A762T) (T7), lacI, Km^r, pBR322*	This study
pET28a-DgARE(A762S)	*His_6-DgARE(A762S) (T7), lacI, Km^r, pBR322*	This study
pET28a-DaSRE	*His_6-DaSRE (T7), lacI, Km^r, pBR322*	Twist Biosciences
pET28a-DaSRE-AE	*His_6-SUMO-DaSRE-AE (T7), lacI, Km^r, pBR322*	Twist Biosciences
pET28a-DaSRE(S801G)	*His_6-DaSRE(S801G) (T7), lacI, Km^r, pBR322*	This study
pET28a-DaSRE(S801T)	*His_6-DaSRE(S801T) (T7), lacI, Km^r, pBR322*	This study
pET28a-DaSRE(S801A)	*His_6-DaSRE(S801A) (T7), lacI, Km^r, pBR322*	This study
pPH149-isc	*E. coli iscSUA-HscBA-Fd (T7), lacI, Cm^r, p15A*	Wecksler et al.[6]

C. Oligonucleotide sequences

Name	*Sequence (5′ to 3′)*
FpTRE1_FP	catcacagcagcggcctggtgccgcgcggcagccatatggtgcgtgcgcaaaagggtagc
FpTRE1_RP	agccggatctcagtggtggtggtggtggtgctcgagctacagtgctccgaattccatgcg
FpTRE2_FP	acagcagcggcctggtgccgcgcggcagccatatgatggcagccaacgagcgcgtctcac

FpTRE2_RP	gcagccggatctcagtggtggtggtggtggtgctcgagtcaatgcaactctgagcgctcg
FpTRE-AE_FP	atattattgaggctcacagagaacagattggtgggatgagccacgaggctcttgtgtttc
FpTRE-AE_RP	cctttcgggctttgttagcagccggatcagcgcgctatcccccgatctgggtatggaatc
pET28a_FP	cctggtgccgcgcggcagccatatg
pET28a_RP	agtggtggtggtggtggtgctcgag
SUMO_bb_FP	cgcgctgatccggctgctaac
SUMO_bb_RP	cccaccaatctgttctctgtgagcc
NStrep_FpTRE2_FP	tatggctagctggagccacccgcagttcgaaaaaggcgccatgatggcagccaacgagcg
NStrep_FpTRE2_RP	ggctttgttagcagccggatcaagcttagttagatatctcaatgcaactctgagcgctcg
FpTRE1_T770G_FP	cgtgtggcgggctatagcgca
FpTRE1_T770G_RP	tgcgctatagcccgccacacg
FpTRE1_T770A_FP	cgtgtggcggcgtatagcgca
FpTRE1_T770A_RP	tgcgctatacgccgccacacg
FpTRE1_C437S_FP	attacaggtagcaactcacct
FpTRE1_C437S_RP	aggtgagttgctacctgtaat
FpTRE1_T770V_FP	cttgttcgtgtggcggtgtatagcgcatacttt
FpTRE1_T770V_RP	aaagtatgcgctatacaccgccacacgaacaag
FpTRE1_T770I_FP	cttgttcgtgtggcgatttatagcgcatacttt
FpTRE1_T770I_RP	aaagtatgcgctataaatcgccacacgaacaag
FpTRE1_T770S_FP	cttgttcgtgtggcgagctatagcgcatacttt
FpTRE1_T770S_RP	aaagtatgcgctatagctcgccacacgaacaag
FpTRE1_T770C_FP	cttgttcgtgtggcgtgctatagcgcatacttt
FpTRE1_T770C_RP	aaagtatgcgctatagcacgccacacgaacaag
DgARE_FP	gcctggtgccgcgcggcagccatatgtctattgatgttgaaagaaagagatttcgtgaag
DgARE_RP	tcagtggtggtggtggtggtgctcgagctatatctcaagttcgctgcgggctataatttc
DaSRE_FP	cacagcagcggcctggtgccgcgcggcagccatatgagtgcaataaatgtcggcacatgg
DaSRE_RP	ggtggtggtgctcgagttaccaagatgtctgttcatttcttctaataatatctatctgac
DgARE_A762G_FP	gtacgggtagctggctatgtggctttattc
DgARE_A762T_FP	gtacgggtagctacctatgtggctttattc
DgARE_A762S_FP	gtacgggtagctagctatgtggctttattc
DgARE_AlaMut_RP	cattaggtcgggatacttttccgggtactt
DaSRE_S801G_FP	cttcttgttcgagttgcaggctataatgca
DaSRE_S801T_FP	cttcttgttcgagttgcaacctataatgca
DaSRE_S801A_FP	cttcttgttcgagttgcagcgtataatgca
DaSRE_SerMut_RP	tcctttatgcttttccggatgtaccattgcatc

D. Codon optimized DNA sequences

D1. Desulfallas geothermicus ARE

ATGTCTATTGATGTGGAACGCAAAAGATTTCGGGAGGCATGTTTGAAAAAATTGCGGCAGCCGAAACTCACTACCGA
ACGCGTTGTGTTAGTGACCGAAGTTCACAAGGAGTCCGAAGGACTGCCCACAATCATGCGGAGAGCTTTAATGCTGA
AGAAAATTCTGTCGAAAATGACAATCTATATCAAGGATTATGAACTCATCGTGGGTGGCCTGGGCCCGGAACCGTTC
TCTGCGCCGATATACCCGGAGTTTTCGTGGCAGTGGGTTCTTGAACAGTTGGACGACTTCAATCGACGCGATGGTGA
TCGGTTTAATGTGTGCAAAGAAGACAAAAAAATTCTGAGTGACTTGCTCCCGTATTGGAGAGGTAAAGCAGTTGAGG
ATGTGGCCCTTGCTGCCATGCCTGAAGAAGTGAAAGCAGCACGAGAAGCGAAACTGATAGCGTTTGAAAATATGCTT
ACAGGCGGTATAGGTCATTACCTCCCAAACTACGAGAAGGTTCTGCGGAAGGGCCTCCGCAATATCTTAGCCGAAAT
AGATGAGAAACAGAAAAAATTAGATCTCACCAATCCCGAGGAATTTGATCAGTACGTGTTCTACAAAGCCGTTAAAA
TAAGTTGTGAAGCTGTTATATATTTTGCAAAACGTTACGCGGATTTAGCCGAGAGCCAGATGTCGTCATGCTCAACC
AAACGTAAAGCGGCCCTGGAAGAAATAGTGCGGGTGTGCAAACGCGTTCCTGCTGAGCCAGCTCAAACCTTTCACGA
AGCATTACAGTCTCTTTGGTTTATTCATCTCATCTGCTATATCAATCAGAATGGTTTAGCAGTCACTCTGGGCCGAA
TGGACCAATATCTTTATCCGTATTATAAGACGGATATTGAGAAAGGAGTAATGGACAAAGAGAAGGTCTTAAGCTTA
CTCGAGAGCTTTTGGATAAAGTGCAACGAAATCATCAAGCTGTATAACAATACAGCAGCGTCATATTATGGCGGCTT
CCCTATAACGCAGGCTCCACAGGTGGGCGGCTTCACCCCGGAGGGCCGAGATGCCACCAACGAGCTGAGTGAACTCA

TTCTGGAAGTCGAAGAACGAGTTAAACTCCCCCAACCAGATATCGGAGTTTTATACACAAAAGAAATGAGCAACGAC
TTCCTGGCAAAAGCTTGCTCACTGATTCCGCATACGATGAAGCCAAAAATCTTCAATGCCCATATCGGCTTGGATAT
TCTGCTTTCTCTGGGCATACCCTTACAGGATGCGAGAAATTACGCATTTGTGGGCTGTGTTGAGAGCTCTGTTCCCG
GAAAGACATGGGGCTGGCACAACGCGGGCTTGATCAACTTTGGTAAGTGTGTTGAATTAGCAATGAACGATGGCATT
GACATGAAATCTGGCCAGCGTTTGGGACCACCTACCGGCAACTTCGAGAGTTTTGGGACGTTTGATGATTTTTTATA
CGCGTACAGAACCCAGGTGGCATCTGCGGTTAGATTACTGGTAACAGCATTACACGTAGTGGAGGATGCTCACCGCA
AAGTACTCCCGCTGCCCTTCGAGTCCGTTCTGATAGACGACTGCTTGGAGAAGGGGCAAGAACTGAATTCAGGCGGT
GCGTGCTATAACTTTACAGGTATCCAGGGTATTGGCCTGGCCACGGCAGCTGACTCCTTAGCTGCGATAAATTCCCA
TATCTTTGAACATAAACGCCTGAAACCTTCTGAACTCCTGAATGCAGTGAAGTCAAATTTCGTGGGCAAAGAAAATA
TCCGTCTTCTGCTTCTCAACGACTCTCCGAAATATGGAAATGACAACGATCAAGTGGATAGCCTGGCCCGCCTGATA
TCACAGCATTATTGCGAAGAGGTTTTTCGTTACCAGAACAGACGCGGTGGCAAATTTATTCCTGGACTGTTCTCAAT
CTCGGCACATGTACCGTTTGGTCAAGGTGTGGTAACGGCCGACGGGCGTAAATGCAATGATCCCCTGTCAGATGCTT
GCTCACCGGCCCAGGGCCGTATTCGACGCGGTCCTACAGCCGTTGCACGGAGCGTGGCAAAGCTGGATCACGTGCAA
GTTGCAAACGGGACGTTATTGAACGTAAAATTTCCAGTCAGCACTCTCAAAGGAGAGGAAAAAATCATTAAATTAGC
TAGCTATGTTAGAACCTTTATGGAATTGGGCGGTTTTCATATACAGGTCAATGTTGTTAACGTAGAACTTCTTCGCG
AAGCGCAGAAATATCCGGAGAAATACCCAGACCTGATGGTTCGTGTAGCCGCATACGTTGCCTTATTCACCCAGTTG
AGCAAGGAAATGCAAGATGAAATCATAGCCCGCAGTGAGTTAGAAATCTAA

D2. Desulfallas geothermicus ARE-AE

ATGAAGGGCCTTATACACTCAATTCAACACTATAGCATTCAAGATGGCCCAGGTATCCGTAGTACAATTTTCTTTAA
AGGGTGTCCGTTACGTTGCCTGTGGTGTGCAAACCCAGACACACAGAAATTTTTCCCCGAATTGACGTTTGACCCGT
TTCTCTGTCCGCCGGAGTGCAAGGAGTGCTTGAAAGTATGCAGTCTTAAAGCCTTGTTTCTGAACGAGAAGGGTAGC
GTTGACTTTTGTAAGGAAAAATGTAATCTTTGTGAGCAATGCCTTCAGGCCTGTTATTCTAAAGCGCTTAAAAAGGT
TGGTACATATATGGGAGTTGAACAGATTCTGGAAGAGGTGGAACGCGAAAGACCGTTCTATGAAACTTCAGGAGGTG
GAGTAACCATTTCGGGAGGAGAACCGTTAATGCAAGGTGACTTTTTAATTGAATTAATTAAGTCTCTTAAAGAGAAA
AACATAAATGTCGCGCTGGACACCTCCGGCTATGGTCCGTGGTCTCTGTTAAAAGAACTTGCCAATCAGGTCGACTG
GTTTTTGTATGATATAAAGCATATGGATAGCATAAAACATAAAGATCTTACTGGCCAGGATAACAAACTCATTCTGG
AGAACCTGAAAAACTTGATCAAACTTAACTCTAATGTGGTTATCCGGTTCCCGTTAATTCCCGGCTATAACGACGAA
CCATTGAATTTAGTTAGCCTGGGGGAATTCATTAAGAACAAAAGTTTCCTGCGCCTGGAAATTCTGCCTTATCATCG
CCTGGGCGCTAGCAAATATAAATTATTCGGCTTAATCTTTCCCTTGTCGCATTTATCCCCGCCTTCTCGTGAGCGCA
TGCAGGAATTAGAAAGATGGTTTAGAAGAATGGGCGTTGCGTGTAAAGTGGTGTGGTAA

D3. Dethiosulfatibacter aminovorans SRE

ATGAGTGCGATCAACGTTGGTACATGGGAAGAAAAGCTGAAGAAATCATATAGCAAGAGAGTTAAAGATGCGATAGA
CCTTACCCTGAGCACGATACCACGGATCGATCTTGAACATGCCTACGTGGAGATGGATATCATTAATGAAAACAAAG
AGGAAGCCCAAGTAATAAAGCGGGCTAGAACATTCGCGAATTACTTGAAGAAGAAGACAATATACATTAACCCTGAT
GAACTGTTGGTGGGTAACATTTCGAAAGGGGTTCGCTACGCACCCTTCGTAGGCGAGTTGTATGTAGACTTCGTAGA
CCAGGAGCTCGACGACCCGGTAATGGACTATGCTATACGGGAGTTCGACAAGCTCATTATCCCAGACGAAACCCGTA
AGGAATTACGCAATGTCATCATACCATTCTTCAAGAAGCACAGTTATCAGAACTTAGTCTTAAACGAGCTGATGGAA
GATTACGTGCGCGAAAAGACCTGCCCTATGGTTTCAAAGACCCCTACGATACCTAATTGTACGAACCTGATGATACA
AACGGATAGCGGTCACCAAGTTCATAATTACAATAAGGTGTTGCAGATCGGCCTGAAGGGAATCCGCGAAGAAGTAT
TATGGCACAAGGAGAAGATCGAGAAGGACTACGTCCTGAACAACAAGCAAAAGCGATTAGACTTCTACGAGGCCGCG
TTAATCTCCATAGACGCAGCGATAGACTATTCCAAGCGCTATAGTAAGCTCGCAAAGGAGCAAGCTGAGGTTGAGAA
GGATGAGAAGCGCAAACAAGAGTTATTGGAGATATCACGGATTTGCGCCAAGGTTCCCGCCGAGCCTGCAGAAACCT
GGCACGAGGCTCTTCAGAGCGTCTTCATGATTTATGTAATTATGTTCTGCGACGTCCGCAATGTGAGCAATGGCTGG
GGCCGTTTTGACCAGTACATGATGCCGTTTTACCAAAAGACGATCCTGGAAGACAAGACAATGTCTCGGGAGTCTGC
CTTAGAGCTTATGGAGTTGTTCTTAGTTAAGGCCAACCAACATGTGGAGCTGTATAATTTTGCCAACACATCAACCC
AGATGGGGTTCGCCTTGGCAACCCAGATAAACATAGGTGGACAAACTCGTACAGGTGAGGACGCGGTTAACGAGGTT
AGCTATCTTGTACTGGACGCTGAGGAGCAGGTGGGGTTGCAACACCCGGATATTGGCATTCGGATTTATGAGGGTAC
GGATGATAAGTTCATACGGAGAGCAACAGAAGTAGTGCGTCTCGGCCGCGGGAAACCGAAGTTCTTCTTTGACAAGA
AGGAAATGGAGTGTCTTAAAGTGGCTTATCCAGAGGCTCCGATTGAGGAGCTGCGCGACTACGTGGCAACCGGCTGC
ACGGAATCTTTCTTACCTCATATAACAATGTGCCATAGCTATTGTTCAATCATCAACGTGCCTAAAGTACTGGAGTT
AACTATTCACAATGGCAAGTGTGCCTTAACTGGCGAACAATTGGGGCCGCAAACGGGTGAACCTCAGTCATTTAAGT
CCATGGAACAGTTCAAGAACGCTTTCGAGGAACAAATGTTCTGGTGGATGGAGTTAGTTGCTAAGTCGGTAACCGTA

CAGATGAACGCACAAGCGGATCATATGCATGCGCCTTTCTCCAGTATCTTGTTAGATGGGCCCATTGATAAGGGTAA
GGACTTAATCGAAGGCGGTTGCTGGGAGAATTCGTTCGGGATATGGTATGCGGGCCTGGCTCACGCAGCAGATTCGT
TATCTGCAGTAGATACACTTGTATACAAGGAGAAGAATATTGAGTGGGATCAACTCCTGGACGCATTGAAGAATAAC
TGGGAAGGTTATGAGGAGTTACGCCAAATGTGTATGAATGACGTGGAGAAGTATGGCAATGACGATGATTACGCTGA
TTCTTTCGCCGCATATGTTATGGACGTATGGTGTGACTCTATTGAATATATTAATGGTAAGAAAGACTTGCTCCCCC
GATATGGCGGCAAGTACGTGTGCAGTAGTATTGTTTCCAGTTCACCAACCGCTCTGGGTGGAATCACGGGTGCCTTA
CCAAACGGTCGGAAGGACGGAGAGCCCCTGTCCGACACCTCTTCTCCTTCAATGGGTGCGGATCGGCTGGGACCGTC
GGCCGTGATCCTCAGTAATGTGAAGCTGCCGATACATCGTAACGCACTGGGGAATTGCTTGAACCAACGCATCTCGC
CTCAGCTCGTCGAAACGGACGAGGATATAAATAGATTCGTATCGTTCATCAAAAGTTGTAGAGACCTGGATATGTTC
GAGATCCAATTTAATATCATATCGACCGACATTTTGCGGGACGCAATGGTCCACCCGGAAAAGCATAAGGGTCTGCT
CGTGCGGGTCGCGTCCTACAATGCCAACTTCACCGACCTTAATGAGGCATGTCAAATTGACATTATAAGAAGAAACG
AGCAAACTTCCTGGTAA

D4. Dethiosulfatibacter aminovorans SRE-AE

ATGTCTACTCTGAATGTGTTTGAGATTTCTAAATTCGCCACCAAGAATGGTCCCGGTCTTCGCACCATGGTCCATCT
GAAAGGTTGTCCTTTAAGCTGTATTTGGTGTTCTACACCTGAAAGTCAGTTAGAGAATCCGCAGATTATGTTTGACC
CGGTAAAATGTATCCAATGCGGTAAATGCATTGAAAAATGTGAGCAGGGCGCCATCACGATTCAGAATGGTGTAACG
ACCCTGTGTTGGGAAAAATGTAAAAATACTCTGGACTGTGTTACAGCCTGTCAGGCAAACGCCTTGACCATTATTGG
TAAACATTATACTTCTGAGGAGCTTGCAAAAATCATTCTGAGAGATAAAGTGTTTTTTAAAAACAGCGATGGCGGAG
TAACATTTAGTGGGGGCGAACCTCTGTATCGTGTAAATGACTCTATGATTGGTCTCTTTAAAATTCTGAAAAGTAAT
GGCATCAGTATAGGCATTGATACCACGGGCTACGTTCCCTGGAAAAATTTGCTCAAAGTCTTACCATTTGTAGATTT
CTTCCTCTGGGATCTGAAACAAATCAATTCACAGAAGCACAAGGAATACACGGGAGTAGATAATAGCATCATCTTTA
CGAATCTGAAAAAAGTTGATAATTATGGAATTGATCTGTACCTGCGTTGCCCGATTATTCCTGGTTGTAACGACGAT
GCTGAGCATATCAACGGTATCTGCAAAGTTGCGAAGCAGCTTGGATCGCTGAAAGAAATTCACATAATTCCTTTGCA
TCATTATGGGACTAATAGATACGCCAAACTGGGCTTAGAATATCCATTAAACAGCGATCTCAAATTGGAAATCAAAA
CGCTGAACTTCATAAAGGAAGTAATGGATAGTTACAAGCTGCCCTATAAAATTATCTAA

D5. Flavonifractor plautii TRE1

ATGGTGCGTGCGCAAAAGGGTAGCACCGCCCGTGTCGCCAAGTGCGCGGAGCGTATCTTTACGCTTCCGGAGATCTG
TATTGAGCGTGAACGTTATTACACCAACTCTTATCAGGAGACAGAGGGTGAGCCACCATGTTTGCGTCAAGCGAAGG
CGTTTGCTAAGACTTTAGATCATATGTCAATCCAGATCTACGATGGCGAACTGATTGTAGGCTGCCCGTGTAGCAAG
GCCCGCGGCGGGGCAGTGCTTCCCGAACTTAATGCGCAATGGCTGCGCGAGGAAGTGGATGATATGAGCGTTCGCCA
GTGGGATCGCTTCCAGCCTCCATCTGCTCGTGAAAAAGAGGAATTGCTTGCATTGTTACCGTATTGGAAGGGCAAGA
GTGCCTATGACCGTTGGAAGAAACGCATTCCAAAATCGATTTTAGAGCTGTACCAGTCTGGATTCATCGGTGGTGTA
ACTTTTTCATCCAACGGTTTTTATCCCTGCCACGTGGCTGTAGACTACGGTATGATCTTACGCCAGGGCTTACAGGC
CCGTTATGAAGAGGTCTTGAAACGTCGTGACGCGTTAGATTTATATCAGATCAAAAACCTTGACAAATATCACTATT
ACACTGCGATGTCAATCTCCCTTGACGCCGTGCGCCGTTTCTCCCTTCGCTATGCGGCACTGGCCCGTCGCATGGCC
GAGGAAGAACGTGACCCGGTGCGTCAAACCGAATTAAAAGAGATTGCACGCATCTGTGCTAAGGTACCGTGGCAAAG
CGCGGAAACCTTTCACGAGGCGGTGCAGTCAGTCTATATGACCTGGGTTGCACTTATCATCGAATCATGGGGTCATG
GAATGACGTTGGGCCGCCCGGACCAGTACCTTATTGACTTTTATCTGCGCGACCGTGCGGCGGGGCGTATTACTCGC
GAAAAAGCGCAGGAGCTGCTTGAATTATGGTTCGTTAAAGTGAACGGTACGGTGACCCTTGACGACCACGATACAGC
TACATGCTTCGCAGGTTTTCCGCAAGCTGTGAATATTACGCTTGGAGGTCAAGATCGCGAGGGAAACTGTGCTGTCA
ATGAATTGACGTACTTATTGATGGCAGCGGAGGAAAAGGTTGGAATGACCGCGGAAGATCTTGTAATTCGCGTTTCT
GAAAACAGCCCTTATGAGTATCTGTTGGCGGCCACGCGCCTTGCCAAAAGTTTGAAAGGCAAACTGAAGTTTGTTTC
GGACCCTGTCGCTATCGCCCAACTGCTTGCCGATGGTTACCCCCTGGAATTGGCCCGTGACTATATTATTACAGGTT
GTAACTCACCTTCGATTCCAGGCATTTCCCTTGACGTACCTGGCGGACTGTTCAATCTGGCGTTCATGCTGGAGTTA
GCCTTGAATAACGGCGTGTCTCGCATGACCGGACGTCAAGTGGGTTTACCAACCGGCGACGCTCGTAAGTTTACAAC
TTATGAGCAGGTATGGGATGCATTCTGTGAGCAGACCCGTTACTTCTTAGGTGCTGCGGTTCTGATGACTAATGCCG
ATCGTGAGATTTTTGCGGAGTTTTTGCCAATTCCGCTGCAGTCGGCGTTGTTTCACGGGCCAATGGAGCGCGGAAAG
GACCTGTTTAACGGGGGGACGGGACGCTACGCACGTCAATCCATCTCTATTGCTGGAGCGCCGAATGTAGGTGATGG
TCTGGCGGCTTTGAAAAAGGTAGTCTTTGAGGACAAACGTTTAACAATGGAACAAGTTGTGGACGCACTGGACGCCA
ATTTCGAAGGATATGATGAGGTGCACTACCTGCTTTCAAAAGCACCCAAGTTCGGTAATGGCATTCCTTATGTTGAC
CAGATCGTTGACCAAGCGCTTATTTTCTGCTCAGATGTATTACACGCGTATATCGGAGTATGTGGTACACCTCTTAT
CACTGCTGCGGCAACGATTACCGCAAACGTGCCACTTGGCTATGGGGTGGGAGCCTTACCAGGGGGGCGTCTTGCAA

ATACTCCACTTGCAGAAGGCGGGATCTCGCCCTATCAGGGCCGCAACCAGAACGGGCCTACTGCCACGATGCGTTCC
GTTGCTGGCCTGACACATACCAAGTTGACGAATGGATCAGTATTGAACATGCGTTTCGATCCTAACGCGTTGAAAGA
CCGCGCAGGCATGGAAAAATTTACAGACCTGTTGCGTGCCTACCTTTTAGGTGGGGGATTCTTCGTACAATTTAACA
TCGTGGACACGGAGACTTTGCGTCAGGCTCAAAAGGAACCTGATCGCTACCGCGATTTACTTGTTCGTGTGGCGACC
TATAGCGCATACTTTGTGGAGCTGAGTCCTGAGATGCAGGAGGATATCATTAACCGCATGGAATTCGGAGCACTGTA
G

D6. Flavonifractor plautii TRE2

ATGATGGCAGCCAACGAGCGCGTCTCACGCTTGCGCGAACGCATGTTGGTAACTCCAGCTATTTGCGTAGAACGTGC
CTATTACATGACAAAATCGTATCAGAAGACGGAGAATCTGCCGACCATCTTACGTCGTGCACATGCTTTAGCTGACA
TTTTGGACCATATGACTATTCGTATCGAGGAAGGCGAGCTTATTGCGGGATGGCAGACTTCCAAGGAGCGTGGTGGC
GCGTTGCTTATTGAAATGCGTTGCGATTGGATCATGGATGAGTTGGATTCGGTTCAATGCCGTGAGTGGGACAAGTA
TCAGCCGTTGTCAGAGGAGGAGAAAGCCATGATCCGTGAAATCGTCCCGTACTGGCAGGGTAAGACGCTGAGTGATT
ATTGGCTGGGCGCGGTTCCTCACTGGGCGGCCAAGCTTGAGAATGTCTTGCAGACAGGTGGATTCTGTCGTAATGGT
CACCATCAGGCACATAGCGTAGGGGACTACGAGTCAATTTTGAAATACGGATTGCGCGCCACATTGGACCTTGTGGA
GAAAAAACTTTCGTCGATTGATCCCTTTAGCCCAGAAGATTTAGACCGTTATCACTTCTATCAGTCGGTAAAGATTG
TGCAAGAAGCAGTGATCCGCCACGCGCACCGTTATGCCGACTTAGGCGAAAAAATGGCGAACGAAGAGCCAGATCCA
GTCCGCCGTGCAGAACTGAAAGAGCTTGCTGCTAACTGCCGCCACGTACCGGAGCACCCCGCACGCACGTTTCGTGA
AGCAATTCAGTCTATCTGGTTGGTTTTCGTACCATTAATGATCGAGTGCTGGGGAGCCGGCATGTCCTTGGGCCGCG
TAGACCAGTATTTATTGCCCTTTTACCAAGCAGACCTGGCTGCTGGACGTATCACTCGCGACGAAGCCCTTGAATTG
GCTTCTTTATTACTTATCAAATTAAATGCCGCCATTAATTTACAAGAAGGATTCCTGTCTGTTGCATTCAGTGGGTA
TCCCGTGATGCAAGGCTTAACAATCGGAGGCATCACGCCCGACGGACGTGATGCCGTTAATGAATTAACCTATTTGT
TTCTTGATGCCGAAGCGGACGTCGGACTGACAGCAGAAGATGTGGTCGTACGTATCTCTCGCTATAACCCGGAGAAA
TTTGTGATTCGCGCATGTGAAGTGGCACGCGATTTACATGGGAAGCTTAAATTCGTCTCGGATGATACGTCCATTCC
CTCGTTGTTACAGATGGGTACTCCCCTGGAGGATGCCCGCGACTATGTAAGTGTCGGCTGCCATAATCCGTCGATCC
CTTATCGCGCCCACGACCCGAATGGGAATGTAGTAAACTGTGGCTTGATGATCGAATTAGCTTTAAATAACGGTGTC
TTCCGTCAAACTGGTGAGCGCATTGGCGCGGAAACGGGTGACCCGCGCTCTTTTACGAGCTTTGACCAGGTACGCGA
AGCGTTCATGATTCAATTTCGTTATCTTATGCGCGCGACGTTTATCTTCAAGAACGCGGATATGCAGATGTTCGGAG
AACACTCGGCCTGCCCACTTTTATCGAGTGTGTATCCGGTTTGCTTAGAAAAGGGGATTGACATCTATCAGGGTGGC
ACCTTCCCGCAACTTTCTCATACGACCGGTTTGGGGTGTCTTGCCGACGTGGGAGATTCATTGGCAGCGATCAAAAA
AGTTGTTTTTGATGACAAGAAGATCACGATGGCCCAATTGTGTGACGCATTAGACGCTAACTTCGAGGGATTCGACG
ATGTACGCCGCTTGTTGAAACAGGCTCCGAAGTTTGGAAATGACGATGATTATGTGGACTTACTTCTTCGCGATGTG
TTACGCGAAATGTGTCACTATATCAAACAATTCAAAACATACAAAAATGCTCGCAACAGCTGTTCTAGCATTACAAT
GACTGCCAATATTCCGTACGGTGCCATTCTGGGCGCAACTCCGGATGGCCGTAAGGCGGGAGAACCTTTAGCAGAAG
GTGGTATCAGTCCACATCAAGGCCGTAACGTATCCGGAGTAACGAGCACTTTCCGTTCAGTTGCAAAGCTGGACCAA
GAACTGTTAGCGAACGGGTCCATTTTGAACATTCGTGTCAGCCCTGAACTTTGTAAAAACGAAAATGGGTTGCGTAA
TTTTGCCCTTATGTTACGTACATTCTGTGAGAATGGAGGGAACCTTGTGCAGTTTAATTTCACATCCAACGAGATGC
TGCGTGAAGCTCAGAAATTCCCGGAGAAATACAAGGACTTGTTGGTACGCGTGGCTACTTATTCCGCGTATTTCACA
GAGCTTTCGCCCGCACTGCAAAATAACATTATCGAGCGCTCAGAGTTGCATTGA

D7. Flavonifractor plautii TRE-AE

ATGAGCCACGAGGCTCTTGTGTTTCATATCATTCATGGGAGTTTTGTCGATGGTTACGGCATTCGTTCTACAGTATT
TCTTAAAGGATGCCCTCTGAAGTGTATTTGGTGCTGCAATCCCGAGGGTCAGAGTTTCCAGCCGGAATTGAAGGTTA
ATCGTGGACAATGTAACGGGTGTGGAAACTGTCTTAATACCTGCTCGCGTAACGCCTTATCGTTGGTAGGAGGGCTT
GTGCAGATTAACCGTGACATCTGTGACGGTTGCATGGAGTGTGTTGAACATTGTTATACTTCGGCCTTAGAACCGTT
TGGCGTTCGCTATACAGCGCACGAGATTTTTGAGATTGTCCGTAGCGATGAAATGTTCTATAAATCTACAGGGGGTG
GCATCACCATCGGAGGCGGCGAATGCACATGCTACCCAGATTTTATGCTGGAACTGACACAACTGTGCCACAACAGC
GGAATCCACGTAGCCGTTGATACTTGTGGATATACAACGTCGTCAAAAGCGCTTGAGGTTTTGAAAGCCGCAGATTT
ACTTCTGTTCGACATTAAGGGTATCGACCCAATGCGTCATAAGAAAAACACTGGGAAATCGAATGAGGTTATCCTTC
AGAATCTTCATCTGCTGAGCGAGATGCGCAAACCGATGATTATCCGTGTACCAGTAATTCCAGGATACAACGACAGT
GACGAAGAGTTAAACGGGATTGCGGACCTTTTGAGCACGTTAAAATCTGTGGAACGCGTAGACATCTTACCAGAACA
TGAATTTGGTCGTATCAAGTACGAGCAATTGGACCTTGAATATCGCCTTAAACCCTGTCCCGTGTCAGCTGAGCGCC
AAGATGCTATCCGCGTAATGTTTGAAGCCAAGGGATTCCATACCCAGATCGGGGGATAG

Table S5. UPLC–MS/MS analysis of standards used for assays.

Standard	***Transition (m/z)***	***Mode***	***Cone (V)***	***Collision (V)***	***Retention (min)***
5′-Deoxyadenosine (5′-dA)	252.0062 > 135.9766	ESI	2	14	0.60
S-Adenosyl-L-homocysteine (SAH)	399.1181 > 250.0088	ESI	2	12	0.47
Methylthioadenosine (MTA)	297.9942 > 162.9676	ESI	62	12	0.95
Adenosine (internal standard)	268.0010 > 135.9765	ESI	4	14	0.60

Table S6. Hyperfine tensors (MHz) of simulated alanyl, serinyl, and threonyl radicals. [a]The ARE simulations employ g =1.999, 2.004, 2.007; isotropic linewidth = 11 MHz; and tabulated hyperfine couplings. [b]The SRE simulations employ g = 2.004, 2.005, 2.006; isotropic linewidth = 11 MHz; and tabulated hyperfine couplings. [c]The TRE simulations employ g = 2.0042, 2.0025, 2.001; isotropic linewidth = 5 MHz; Hstrain = [10, 10, 20] MHz (inhomogeneous line broadening). Q-band EPR spectra establish that the appearance of the low-field shoulder in the X-band spectra (**Figure 2B**) arises from an interplay of g-anisotropy and ^{1}H hyperfine couplings. Those couplings are *not* well-defined, but representative values are given as tabulated hyperfine couplings. The simulation g-values for the X-band EPR spectra collected at MIT are adjusted based on Q-band spectra from NU.

	A_1	A_2	A_3
	***Dg*ARE[a]**		
C_β–$^{1}H_a$	40	40	40
C_β–$^{1}H_b$	40	40	40
C_β–$^{1}H_c$	40	40	40
	***Da*SRE[b]**		
C_β–$^{1}H_a$	48	49	49
C_β–$^{1}H_b$	86	84	84
	***Fp*TRE1/2[c]**		
C_β–$^{1}H_a$	19	6	6
^{14}N–H	4	1	5

General materials and methods

All chemicals and solvents were purchased from Sigma–Aldrich, except where otherwise noted. *Desulfallas geothermicus* DSM 3669 and *Dethiosulfatibacter aminovorans* DSM 17477 genomic DNA (gDNA) were purchased from DSMZ. Luria-Bertani Lennox (LB) medium was purchased from Alfa Aesar. DNA sequencing results were analyzed with Benchling (Benchling, Inc.). Multiple sequence alignments were analyzed with Geneious Pro 11.0.4 (Biomatters)[7] or Clustal Omega.[8] All restriction enzymes, ligases, polymerases, PCR mixes, and Gibson Assembly mixes were obtained from New England Biolabs (NEB). SDS-PAGE (4–15% Mini-PROTEAN TGX gel, Bio-Rad) was routinely used to visualize fractions from protein purifications following staining (InstantBlue Coomassie Protein Stain, Abcam). Isopropyl β-D-1-thiogalactopyranoside (IPTG) was obtained from Teknova. Ni-NTA resin was obtained from Qiagen. Dithiothreitol (DTT) was purchased from VWR International. *S*-adenosyl-L-methionine (SAM) was purchased from Sigma-Aldrich as a *p*-toluenesulfonate salt. Solvents used for LC–MS were B&J Brand high-purity solvents (Honeywell Burdick & Jackson).

Samples were made anaerobic as follows. Solids were brought into anaerobic chambers (MBraun – atmosphere of N_2 and <0.1 ppm O_2; Coy Labs – atmosphere of 97% N_2 and 3% H_2) in perforated 1.7 mL microcentrifuge tubes. Protein solutions with volumes greater than 1 mL were made anaerobic on a Schlenk line with 20 cycles of evacuation on vacuum followed by filling with argon or nitrogen. Solutions (<50 mL) were made anaerobic by bubbling argon or nitrogen through the liquid for 20 min. Solutions (≥50 mL) were made anaerobic on a Schlenk line with 3 cycles of evacuation on vacuum followed by filling with argon or nitrogen for 30 min each while stirring.

Bacterial strains

E. coli Top10 (Invitrogen) was routinely used for DNA construction and either BL21(DE3) (Invitrogen), BL21(DE3) *ΔiscR,*[9] or EH1(DE3)[10] was used for heterologous production of proteins for biochemical experiments.

To prepare chemically competent cells for plasmid transformations, the appropriate *E. coli* strain was cultured from a single colony in 100 mL of LB broth, harvested at OD_{600} of 0.4–0.5, and resuspended in 10 mL of sterile Transformation-Storage Solution (10% *w*/*v* PEG 6000, 5% *v*/*v* DMSO, 20 mM $MgCl_2$ in LB broth). 100 μL aliquots were flash frozen in liquid nitrogen and stored at –80 °C for later use. Plasmid transformations were performed by adding one or more plasmid (100 ng), 20 μL 5× KCM solution (0.5 M KCl, 0.15 M $CaCl_2$, 0.25 M $MgCl_2$), and 80 μL of sterile water to a thawed competent cell aliquot of the appropriate strain. After 10 min incubation on ice, cells were heat shocked at 42 °C for 90 s, diluted to 1 mL with LB broth, and incubated at 37 °C for 1 h before plating with sterile beads on LB agar containing appropriate antibiotics.

Plasmid construction

Plasmid construction was carried out using standard molecular biology techniques. Descriptions of strains and plasmids are listed in **Table S4AB**. PCR amplifications were carried out with Q5 High-Fidelity or Phusion High-Fidelity polymerase, following manufacturer instructions. Primers (Sigma–Aldrich) are listed in **Table S4C**. Typical PCR reactions (total volume 50 μL) contained 10 ng template, 0.6 μM forward primer, 0.6 μM reverse primer, optional 1% DMSO, and either Q5-HF or 2× Phusion-HF Master Mix (25 μL) (NEB). PCR parameters were as follows: Initial denaturation (98 °C for 30 s), 30 cycles of denaturation (98 °C for 10 s), annealing (55 °C for 30 s), extension (72 °C for 90 s), and a final extension (72 °C for 5 min). All PCR amplifications were analyzed by 1% agarose gel electrophoresis with ethidium bromide staining in 1× tris-acetate-EDTA (TAE) buffer. PCR products and digested DNA were purified using Zymoclean DNA Clean & Concentrator Kit or Gel DNA Recovery Kit (Zymo Research). Constructs were assembled using Gibson Assembly[11] (NEB), following manufacturer instructions. Constructs were verified by sequencing (Eton Bioscience or Genewiz). DNA was purified using E.Z.N.A. Plasmid MiniKit (Omega Bio-tek) and

transformed into chemically competent *E. coli* BL21(DE3) cells. Transformed cells were stored at –80 °C as frozen LB/glycerol stocks.

Construction of plasmids for the expression of FpTRE1, FpTRE2, and FpTRE-AE. Codon optimized *Fp*TRE1 (NCBI ID: MCB7360671.1), *Fp*TRE2 (NCBI ID: MCB7360675.1), and *Fp*TRE-AE (NCBI ID: MCB7360676.1) genes were synthesized (Twist Bioscience). Codon optimized nucleotide sequences can be found in **Table S4D**. pET28a-*Fp*TRE1 and pET28a-*Fp*TRE2 were constructed by amplifying the *Fp*TRE1 and *Fp*TRE2 genes using primer sets FpTRE1_FP & FpTRE1_RP and FpTRE2_FP & FpTRE2_RP, respectively. These fragments were inserted into pET28a digested with NdeI and XhoI using Gibson assembly.[11] pET-FpTRE-AE was constructed by amplifying the *Fp*TRE-AE gene using primer set FpTRE-AE_FP & FpTRE-AE_RP. The pET backbone[12] containing the N-His$_6$-SUMO tag was amplified using primer set SUMO_bb_FP & SUMO_bb_RP and ligated with the appropriate PCR fragment using Gibson assembly.[11]

Construction of plasmids for the expression of DgARE, DgARE-AE, DaSRE, and DaSRE-AE. pET28a-DgARE, pET28a-DgARE-AE, pET28a-DaSRE, and pET28a-DaSRE-AE were synthesized to encode codon optimized *Dg*ARE (UniProt ID: A0A1I6D5X9), *Dg*ARE-AE (UniProt ID: A0A1I6D686), *Da*SRE (UniProt ID: A0A1M6EMU2), and *Da*SRE-AE (UniProt ID: A0A1M6EN36) respectively (Twist Bioscience). Codon optimized nucleotide sequences can be found in **Table S4D**.

Site-directed mutagenesis of FpTRE1, FpTRE2, DgARE, and DaSRE. Point mutations were introduced through either three-piece Gibson Assembly[11] or KLD Assembly (NEB), depending on cloning difficulties. For *Dg*ARE and *Da*SRE, point variants were derived from the non-codon-optimized genes. The *Dg*ARE (UniProt ID: A0A1I6D5X9) gene was amplified from *Desulfallas geothermicus* DSM 3669 (DSMZ, Braunschweig, Germany) using primer set DgARE_FP & DgARE_RP. The *Da*SRE (UniProt ID: A0A1M6EMU2) gene was amplified from *Dethiosulfatibacter aminovorans* DSM 17477 (DSMZ) using primer set DaSRE_FP & DaSRE_RP.

For a typical Gibson Assembly procedure [*e.g.,* pET28a-FpTRE1(T770G)], the *Fp*TRE1 gene was amplified in two segments using primer sets pET28a_FP & FpTRE1_T770G_RP and FpTRE1_T770G_FP & pET28a_RP. The two segments were inserted into pET28a digested with NdeI and XhoI using the Gibson Assembly.[11] For a typical KLD Assembly procedure [*e.g.,* pET28a-FpTRE2(T764G)], the entire pET28a-FpTRE2 plasmid was amplified using primer set FpTRE2_T764G_FP & FpTRE2_ThrMut_RP. The final plasmid was assembled using the KLD enzyme mix (NEB). Other *Fp*TRE1 (T770A, C437S, C437S/T770G, C437S/T770A, T770V, T770I, T770S, T770C), *Fp*TRE2 (T764A, C431S, C431S/T764G, C431S/T764A, T764V, T764I, T764S, T764C), *Dg*ARE (A762G, A762T, A762S) and *Da*SRE (S801G, S801T, S801A) mutant constructs were constructed using a similar strategy.

Expression and purification of heterologously expressed enzymes

Expression and purification of His$_6$-tagged DgARE, DaSRE, FpTRE1, and FpTRE2 enzymes. *Dg*ARE, *Da*SRE, *Fp*TRE1, *Fp*TRE2, and corresponding variants were expressed and purified in a similar fashion. Chemically competent *E. coli* BL21(DE3) was transformed with the appropriate pET28a expression vector. An overnight culture of the expression strain was grown in LB containing 50 μg mL^{-1} kanamycin (Km) starting from a colony or glycerol stock (37 °C, 180 rpm). 2 L of LB media in a 2.8 L Erlenmeyer baffled flask with 50 μg mL^{-1} of Km was inoculated with 40 mL of starter culture (2%). The culture was grown at 37 °C at 180 rpm to OD$_{600}$ = 0.5–0.6, at which point cultures were cooled on ice for 20 min, induced with 500 μM IPTG, and grown overnight at 16 °C at 180 rpm. Cell pellets were harvested by centrifugation at 6,730 × *g* for 10 min at 4 °C, flash frozen in N_2 (*l*), and stored at –80 °C.

All subsequent steps were performed at 4 °C unless otherwise specified. Frozen cell pellets were thawed and resuspended at 5 mL per g cell paste with Buffer A1 (44 mM $K_3(PO_4)_2$, 500 mM NaCl, 20% glycerol, pH 7.2). Cells were lysed by three passages through a cell disrupter (Avestin EmulsiFlex-C3) at 10,000 psi

or by sonicating with a ½ inch horn at 25% amplitude for 4 min (2.5 s on followed by 5 s off) while being kept in an ice bath. Lysate was centrifuged at 18,000 × *g* for 30 min at 4 °C to separate soluble and insoluble fractions. The soluble lysate was loaded onto a Ni-NTA column (3 mL resuspended resin per 2 L expressed pre-equilibrated with Buffer A1) by gravity flow. The column was washed with 3 column volumes (c.v.) of 95:5 Buffer A1:Buffer A2 (44 mM $K_3(PO_4)_2$, 500 mM NaCl, 20% glycerol, 500 mM imidazole, pH 7.2), 3 c.v. of 90:10 Buffer A1:Buffer A2, and 3 c.v. of 85:15 Buffer A1:Buffer A2 until no protein was detectable by Bradford Protein Assay (Bio-Rad). Protein was eluted from the column with Buffer A2 in 5 mL fractions. Fractions containing protein, as detected by Bradford and SDS-PAGE, were combined and concentrated using an Ultra-15 Centrifugal Filter with a 50 kDa MWCO (Amicon). To buffer exchange, the protein was passed through a PD-10 desalting column packed with Sephadex G-25 resin (Cytiva) equilibrated with Buffer A3 (25 mM HEPES, 200 mM NaCl, 20% glycerol, pH 8). A spin concentrator was used to further concentrate the eluted to a protein concentration of ~350 μM. Protein concentrations were estimated using a NanoDrop 2000 UV–vis Spectrophotometer (Thermo Scientific) and ε_{280nm} calculated by ExPASY ProtParam[13] as follows: *Dg*ARE and all point variants (ε_{280} = 83,200 M^{-1} cm^{-1}), *Da*SRE and all point variants (ε_{280} = 115,170 M^{-1} cm^{-1}), *Fp*TRE1 and all point variants (ε_{280} = 103,140 M^{-1} cm^{-1}), *Fp*TRE2 and all point variants (ε_{280} = 85,720 M^{-1} cm^{-1}). Typical yields of *Dg*ARE is 5 mg L^{-1}, of *Da*SRE is 5 mg L^{-1}, of *Fp*TRE1 is 0.5–13 mg L^{-1}, and of *Fp*TRE2 is 2 mg L^{-1}. The concentrated protein solution was made anaerobic on a Schlenk line with 20 cycles of evacuation on vacuum and filling with nitrogen and brought into an anaerobic chamber at 4 °C containing 97% N_2 and 3% H_2 (Coy Labs), aliquoted into 0.5 mL cryogenic vials with o-rings, flash frozen in N_2 (*l*), and stored in a N_2 (*l*) storage dewar.

Determination of DgARE, DaSRE, FpTRE1, FpTRE2 oligomeric state. Size-exclusion chromatography was conducted using a BioLogic DuoFlow FPLC (Bio-Rad). AARE (10 mg) was loaded onto a Superdex 200 increase 10/300 GL size exclusion column (Cytiva 28990944) pre-equilibrated in 25 mM Tris, pH 8, 200 mM NaCl, and 1 mM DTT. Calibration was conducted using the Bio-Rad gel filtration standard (cat. no. 1511901) and a second order line of best fit.

Expression and purification of His

Expression and purification of His_6-tagged DgARE-AE, DaSRE-AE, and FpTRE-AE enzymes. *Dg*ARE-AE, *Da*SRE-AE, and *Fp*TRE-AE were overexpressed and purified based on a previously reported protocol.[14] Chemically competent *E. coli* BL21(DE3) Δ*iscR* was co-transformed with the appropriate expression vector (pET for His_6-SUMO-tagged proteins, pET28a for His_6-tagged proteins) and pPH149 (vector with IPTG-inducible *E. coli iscSUA-HscBA-Fd* genes for FeS cluster biosynthesis).[6]

An overnight culture of the appropriate strain was grown in LB media containing 50 μg mL^{-1} kanamycin (Km) (pET28a) or 100 μg mL^{-1} ampicillin (Amp) (pET) and 50 μg mL^{-1} chloramphenicol (Cm) (pPH149), starting from a colony or glycerol stock. 2 L of LB media supplemented with 20 mM $MgCl_2 \cdot 6\ H_2O$ in a lightly screw-capped 2.8 L baffled Fernbach flask (Corning) was allowed to equilibrate overnight at 37 °C. Just prior to inoculation with 40 mL of starter culture (2%), the media was supplemented with 20 mM glucose and either 50 μg mL^{-1} of Km or 100 μg mL^{-1} of Amp and 50 μg mL^{-1} Cm. The culture was grown at 37 °C at 180 rpm to OD_{600} = 0.5–0.6, at which point 2 mM L-Cys • HCl, 500 μM $(NH_4)_2Fe(II)(SO_4)_2 \cdot 6\ H_2O$, and 50 mM sodium fumarate dibasic were added. Cultures were cooled on ice for 20 min and induced with 500 μM IPTG. The flask was immediately tightly sealed with a septa and screw cap before growing overnight at 16 °C at 180 rpm. Cell pellets were harvested aerobically by centrifugation at 6,730 × *g* for 10 min at 4 °C, flash frozen in N_2 (*l*), and stored at –80 °C.

All subsequent purification steps were performed in an anaerobic chamber at 4 °C containing 97% N_2 and 3% H_2 (Coy Labs). Frozen cell pellets were thawed, resuspended at 5 mL per g cell paste with anoxic Buffer B1 (25 mM HEPES, 200 mM NaCl, pH 8) (all AEs) supplemented with 10 mg L^{-1} of chicken egg lysozyme, 10 mg L^{-1} DNase I, 10 mM $MgCl_2 \cdot 6\ H_2O$, 1 mM β-mercaptoethanol (BME), and SIGMA*FAST* Protease Inhibitor Cocktail Tablets, EDTA-Free. Cells were lysed by sonicating with a ½ inch horn at 30% amplitude for 6 min (2.5 s on followed by 5 s off). The resulting suspension became dark grey during lysis. Lysate was transferred into a 50 mL centrifuge tube, taken out of the anaerobic chamber, and centrifuged at 18,000 × *g* for 30 min at 4 °C to separate soluble and insoluble fractions before being brought back into the anaerobic

chamber. The soluble lysate was loaded onto a Ni-NTA column (3 mL resuspended resin per 2 L expressed, pre-equilibrated with anoxic Buffer B1) by gravity flow. The column was washed with 10 c.v. of 90% Buffer B1, 10% Buffer B2 (25 mM HEPES, 200 mM NaCl, 250 mM imidazole, pH 8), 5 c.v. of 80% Buffer B1, 20% Buffer B2, and 5 c.v. of 70% Buffer B1, 30% Buffer B2. Protein was eluted from the column with Buffer B2 in 5 mL fractions. Brown elution fractions containing protein, as detected by Bradford and SDS-PAGE, were combined and concentrated using an Ultra-15 Centrifugal Filter with a 10 kDa MWCO (Amicon). To buffer exchange, the protein was passed through a PD-10 desalting column packed with Sephadex G-25 resin (Cytiva) equilibrated with Buffer B3 (25 mM HEPES, 200 mM NaCl, 10% glycerol, pH 8). A spin concentrator was used to further concentrate the eluted to a protein concentration of ~200 μM. Protein concentrations were estimated using a NanoDrop 2000 UV–vis Spectrophotometer (Thermo Scientific) and ε_{280nm} calculated by ExPASY ProtParam[13] as follows: *Dg*ARE-AE (ε = 42,400 M^{-1} cm^{-1}), *Da*SRE-AE (ε = 39,880 M^{-1} cm^{-1}), and *Fp*TRE-AE (ε = 20,400 M^{-1} cm^{-1}). Typical yields of *Dg*ARE-AE is 1 mg L^{-1}, of *Da*SRE-AE is 1 mg L^{-1}, and of *Fp*TRE-AE is 3 mg L^{-1}.

Radical quantitation by electron paramagnetic resonance (EPR) spectroscopy

Wild-type (WT) *Fp*TRE1, *Fp*TRE2, *Dg*ARE, *Da*SRE and variants were prepared for EPR spectroscopy as follows. All assay concentrations are final concentrations. Anoxic AARE and AARE-AE aliquots were brought into an anaerobic chamber containing either 97% N_2 and 3% H_2 (Coy Labs) or N_2 and < 0.1 ppm O_2 at 22 °C (Mbraun). Reaction mixtures contained 10 mM DTT, 500 μM SAM, 50 or 100 μM AARE-AE, 50 or 100 μM AARE (total monomer concentration), and 250 or 500 μM sodium dithionite (NaDT) in buffer (50 mM HEPES, 50 mM NaCl, pH 8.0). The samples were reduced under ambient light for 2 h. The entire 250 μL reaction mixture was used for analysis.

Perpendicular mode X-band EPR spectra were recorded on a Bruker EMX-Plus EPR instrument equipped with a Bruker/ColdEdge 4 K waveguide cryogen-free cryostat set at 77 K (Massachusetts Institute of Technology [MIT]), Bruker ESP 300 spectrometer equipped with an Oxford Instruments ESR 910 continuous helium flow cryostat (Northwestern University [NU]), or Bruker EMX EPR spectrometer equipped with a Bruker/Cold Edge (Sumitomo Cryogenics) 10 K waveguide cryogen-free system with an Oxford MercuryiTC controller unit and helium Stinger recirculating unit (Sumitomo Cryogenics, ColdEdge Technologies) (Montana State University [MSU]).

All samples were loaded into EPR tubes with 4 mm outer diameter and 8″ length (Wilmad LabGlass, 734-LPV-7), sealed, and frozen in N_2 (*l*). Data acquisition was performed with Xepr software (Bruker). The magnetic field was calibrated with an external standard of $K_2(SO_3)_2NO$ (Frémy) [g_x = 2.00785, g_y = 2.00590, g_z = 2.00265, A_x = 5.5 G, A_y = 5.0 G, A_z = 28.7 G].[15] The experimental spectra for the glycyl, alanyl, serinyl, and threonyl radicals were modeled with EasySpin (Version 5.2.33)[16] for MATLAB (MathWorks) to obtain *g*-values, hyperfine coupling constants, and line widths. Spin concentration measurements were performed by numerically calculating the double integral of the simulated spectra and comparing the area with that of a Frémy standard. This standard was prepared before each set of EPR measurements by dissolving solid Frémy under anaerobic conditions in anoxic 20 mM Tris, 100 mM KCl, pH 7.5 and diluting to a final concentration of 25 μM. To account for any decomposition during dissolution, the concentration was measured at 248 nm (ε = 1,690 M^{-1} cm^{-1}) using a NanoDrop 2000 UV–vis Spectrophotometer.[17]

X-band EPR spectra at MIT were recorded under the following conditions: temperature, 10–100 K; center field, 3350 G; sweep width, 200 G; microwave power, 1.262 μW; microwave frequency, 9.37 GHz; modulation amplitude, 4 G; modulation frequency, 100 kHz; time constant, 0.01 ms; conversion time, 20.12 ms; sweep time, 10.06 s; receiver gain, 52 dB. X-band EPR spectra recorded at NU employed a Bruker ESP 300 spectrometer equipped with an Oxford Instrument ESR 900 continuous-liquid-helium-flow system: T = 40 K; microwave frequency, 9.371 GHz; modulation amplitude, 10 G. Q-band EPR spectra were recorded in a Bruker EMX spectrometer as: T = 40 K; microwave frequency, 34.01 GHz; modulation amplitude, 5 G. Q-band CW ENDOR spectra were recorded as described previously[18]: T = 2 K; microwave frequency, 34.94 GHz; modulation amplitude, 2 G; time constant, 32 ms; scan direction, forward; scan

speed, 0.5 MHz s^{-1}. X-band EPR spectra at MSU were recorded at 9.38 GHz and 10–77 K using 1.0–5.0 G modulation amplitude and 1 μW–3 mW power.

UV–vis assays of FeS cluster reduction

To obtain UV–vis absorption spectra, *Dg*ARE-AE, *Da*SRE-AE, and *Fp*TRE-AE was diluted to 25 μM with anoxic buffer (50 mM HEPES, 50 mM NaCl, pH 8.0) inside of an anaerobic chamber containing 97% N_2 and 3% H_2 (Coy Labs). The absorbance of the solution was measured from 200 nm to 1200 nm in a septa-sealed Ultra-Micro Cell quartz cuvette (Hellma) using a Cary 8454 UV–vis Diode Array System (Agilent). To obtain a spectrum for the reduced protein, 100 μM NaDT was added with a gas-tight syringe before the absorbance was measured at 1, 4, 10, 25, and 30 min.

Generation of multiple sequence alignment and phylogenetic tree

The initial multiple sequence alignment (MSA) of the InterPro IP004184 (pyruvate formate-lyase domain) family was created using the default parameters of FAMSA (fast and accurate multiple sequence alignment).[19] To de-replicate and prune the number the sequences, we used the Enzyme Function Initiative-Enzyme Similarity Tool (EFI-EST)[20] to only keep sequences that shared <40% amino acid identity to one another. To this list, we added back biochemically characterized GREs (Uniprot IDs: A9J4K4, E5Y7I4, E5Y378, Q8GEZ8, C9XIS5, C9YHW1, Q18CP5, Q84F16, A0A031WDE4, A0A316Q2B4, Q46266, P37836, Q38HX4, A0A0E3M8P3, B8J0I2, Q30W70, Q312S2, B8J0R1, K0ND30, Q2YV53, H1BAW2, P28903, P09373, P32674, P42632, P75793, P07071, P43753, A0A096ZNX3, A0A318FL05, O32797, O32799, D2XBH8, A0A100YWM3, A0A100YXA1, E1QXZ2, AVQ67926, AVQ67923, A3DCR3, A9KN53, I3VT07, Q21A60, Q1A666, Q2FK44, Q5HJF4, Q6GK90, Q5HKH9, Q8CTX6, Q59934, O87943) and the newly identified AARE sequences found in NCBI and UniProt databases. These 2,252 amino acid sequences were aligned using HMMER (v3.3.2)[21] against the pyruvate formate lyase-like profile (PF02901) using the default settings. This final alignment was used to construct a maximum-likelihood phylogenetic tree using IQ-TREE2 with automated ModelFinder[22] and 1000 ultrafast bootstraps.[23] Results were visualized in Geneious Pro 11.0.4 (Biomatters)[7] and Jalview 16:54:56.[24]

Generation of predicted protein structures

Predicted structures were generated with AlphaFold2 with MMseqs2 on the Google ColabFold server (version 1.5.2).[25,26] When possible, proteins were modeled as multimers (homodimer/heterodimer). Structures were visualized and compared in Pymol (version 2.5.4). The short peptide mimics were modeled into AARE-AEs by superimposing the AARE-AE structure to that of PFL-AE (PDB accession code 3CB8). The catalytic Gly of the PFL-peptide was substituted to Ala, Ser, or Thr by using the Mutagenesis Wizard in Pymol.

UPLC–MS/MS assays for detecting end-point SAM cleavage products

LC–MS samples were prepared by combining 10 mM DTT, 500 μM SAM, 50 or 100 μM AARE-AE, 50 or 100 μM AARE (total monomer concentration), and 250 or 500 μM NaDT in buffer (50 mM HEPES, 50 mM NaCl, pH 8.0 in an anaerobic chamber containing N_2 and < 0.1 ppm O_2 at 22 °C (Mbraun) or 3% H_2 and 97% N_2 (Coy Labs). After incubating at room temperature for 2.5 h, the reaction mixtures were taken out of the anaerobic chamber and quenched with 5 equivalents of LC–MS grade acetonitrile (ACN) containing 50 μM adenosine internal standard. The samples were place in a –20 °C freezer for 30 min, centrifuged at 3,220 × *g* for 10 min, 4 °C on a tabletop swinging bucket centrifuge, diluted 4-fold into H_2O, and then diluted a further 20-fold into H_2O.

Simultaneous analysis of SAM, 5′-deoxyadenosine (5′-dA), *S*-adenosyl-L-homocysteine (SAH), methylthioadenosine (MTA), and adenosine was carried out by ultra-performance liquid chromatography tandem mass spectrometry (UPLC–MS/MS). Liquid chromatography was conducted using a Waters

Acquity UPLC H-Class System (Waters Corporation). 1 μL of each sample was injected onto an Acquity UPLC BEH C18 1.7 μm (2.1 × 50 mm) column (Waters Corporation). The flow rate was 0.8 mL min^{-1} using mobile phase A = 0.1% formic acid in H_2O and mobile phase B = 0.1% formic acid in ACN. The column temperature was maintained at 40 °C. The following gradient was applied: 0–1 min at 0–33% B isocratic, 1.0–1.5 min at 33–100% B, 1.5–2.0 min at 100% B isocratic, 2.0–2.1 min at 100–0% B, 2.1–2.8 min at 0% B isocratic. The first 0.4 min of the run was diverted to waste. MS detection was performed with a Waters Xevo TQ-S (Waters Corporation) instrument with electron spray ionization in positive mode (ESI$^+$) (capillary voltage, 0.7 kV; cone voltage, 15 V; source offset voltage, 50 V; desolvation temperature, 500 °C; desolvation gas flow, 1000 L h^{-1}; cone gas flow, 150 L h^{-1}; nebulizer, 7.0 bar). Conditions for tandem MS/MS were optimized using authentic standards. See **Table S5** for specific detection parameters. Standards of 5′-dA, SAH, and MTA were prepared ranging from 50–600 μM in H_2O, in triplicate.

Analysis of metagenomic and metatranscriptomic data

AARE and GRE homologs were compiled to generate a DIAMOND[27] protein database. The negative control set consisted of the following sequences: IAD (Uniprot IDs: A0A0E3M8P3, A0A1A6ASB6, A0A166SJU7, A0A2U8DR13, A0A3D3W7X7, X8HS46, E1QXZ2, F0Z0V1, A0A100YXA1, A0A3E2WJG0, A0A174H1C3 R7D0K5, B1BA70, A0A0L7NFY0), HPAD (Uniprot IDs: C9XIS5, Q38HX4), PFL (Uniprot IDs: A8HMX2, P09373, A3DCR3, I3VT07), TdcE (Uniprot ID: P42632), CutC (Uniprot IDs: Q30W70, B8J0I2, D7ZDQ9), GD (Uniprot ID: Q8GEZ8), HypD (Uniprot ID: T4P1Y6), IslA (Uniprot ID: E5Y378, Q312S2), PD (Uniprot IDs: A9KN53, Q1A666, Q21A60), BSS (Uniprot ID: O87943), HBSS (Uniprot ID: K0ND30), IBSS (Uniprot ID: A0A096ZNX3), MASS (Uniprot ID: A9J4K4), NMSS (Uniprot ID: D2XBH8), NrdD (Uniprot ID: P28903). All computations were run on the FASRC Odyssey/Cannon cluster supported by the FAS Division of Science Research Computing Group at Harvard University. A blastx DIAMOND[27] (e-value, 0.0001; percent amino acid identity, 50%) search was performed using the raw shotgun MGX or MTX sequencing reads against the custom database. If the highest sequence identity hit for the read to an AARE protein was greater than or equal to that of a negative control protein and the % aa ID to the AARE protein was >90%, then that read was considered a positive hit for an *AARE* gene or transcript. The stringent 90% cutoff value was used as this ensured reads mapping to the Gly fingerprint motif retained the non-Gly residue. The cutoff for PFL, HPAD, and CutC was a less stringent 62%. Positive hits for each metagenome sample were aggregated and normalized for sequencing depth and gene length and reported as reads per kilobase million (RPKM). This is broken down in the following equation:

$$RPKM = \frac{Reads\ Per\ Million\ (RPM)}{gene\ length\ [kB]} = \frac{\frac{reads}{total\ reads}\ /\ 10^6}{gene\ length\ [kB]}$$

For MGX data, to compare between samples, RPKM was converted to “copies per microbial genome” (CPMG) with the following equation:

$$Copies\ per\ Microbial\ Genome = RPKM \times Average\ Genome\ Size\ (AGS) \times 10^{-9}$$

The factor of 10^{-9} arises because of unit conversions.[5] Average genome size (AGS) for each sample was calculated using MicrobeCensus.[28]

Supplemental references

(1) Abu-Ali, G. S.; Mehta, R. S.; Lloyd-Price, J.; Mallick, H.; Branck, T.; Ivey, K. L.; Drew, D. A.; Dulong, C.; Rimm, E.; Izard, J.; et al. Metatranscriptome of human faecal microbial communities in a cohort of adult men. *Nat. Microbiol.* **2018**, *3*, 356–366. DOI: 10.1038/s41564-017-0084-4.

(2) Lloyd-Price, J.; Arze, C.; Ananthakrishnan, A. N.; Schirmer, M.; Avila-Pacheco, J.; Poon, T. W.; Andrews, E.; Ajami, N. J.; Bonham, K. S.; Brislawn, C. J.; et al. Multi-omics of the gut microbial ecosystem in inflammatory bowel diseases. *Nature* **2019**, *569*, 655–662. DOI: 10.1038/s41586-019-1237-9.

(3) Schirmer, M.; Franzosa, E. A.; Lloyd-Price, J.; McIver, L. J.; Schwager, R.; Poon, T. W.; Ananthakrishnan, A. N.; Andrews, E.; Barron, G.; Lake, K.; et al. Dynamics of metatranscription in the inflammatory bowel disease gut microbiome. *Nat. Microbiol.* **2018**, *3*, 337–346. DOI: 10.1038/s41564-017-0089-z.

(4) Beller, H. R.; Rodrigues, A. V.; Zargar, K.; Wu, Y.-W.; Saini, A. K.; Saville, R. M.; Pereira, J. H.; Adams, P. D.; Tringe, S. G.; Petzold, C. J.; et al. Discovery of enzymes for toluene synthesis from anoxic microbial communities. *Nat. Chem. Biol.* **2018**, *14*, 451–457. DOI: 10.1038/s41589-018-0017-4.

(5) Levin, B. J.; Huang, Y. Y.; Peck, S. C.; Wei, Y.; Martínez-del Campo, A.; Marks, J. A.; Franzosa, E. A.; Huttenhower, C.; Balskus, E. P. A prominent glycyl radical enzyme in human gut microbiomes metabolizes *trans*-4-hydroxy-L-proline. *Science* **2017**, *355*, eaai8386. DOI: 10.1126/science.aai8386.

(6) Wecksler, S. R.; Stoll, S.; Tran, H.; Magnusson, O. T.; Wu, S.-P.; King, D.; Britt, R. D.; Klinman, J. P. Pyrroloquinoline quinone biogenesis: demonstration that PqqE from *Klebsiella pneumoniae* is a radical *S*-adenosyl-L-methionine enzyme. *Biochemistry* **2009**, *48* (42), 10151–10161. DOI: 10.1021/bi900918b.

(7) Kearse, M.; Moir, R.; Wilson, A.; Stones-Havas, S.; Cheung, M.; Sturrock, S.; Buxton, S.; Cooper, A.; Markowitz, S.; Duran, C.; et al. Geneious Basic: an integrated and extendable desktop software platform for the organization and analysis of sequence data. *Bioinformatics* **2012**, *28*, 1647–1649. DOI: 10.1093/bioinformatics/bts199.

(8) Sievers, F.; Wilm, A.; Dineen, D.; Gibson, T. J.; Karplus, K.; Li, W.; Lopez, R.; McWilliam, H.; Remmert, M.; Soding, J.; et al. Fast, scalable generation of high-quality protein multiple sequence alignments using Clustal Omega. *Molecular Systems Biology* **2014**, *7*, 539–539. DOI: 10.1038/msb.2011.75.

(9) Peck, S. C.; Denger, K.; Burrichter, A.; Irwin, S. M.; Balskus, E. P.; Schleheck, D. A glycyl radical enzyme enables hydrogen sulfide production by the human intestinal bacterium *Bilophila wadsworthia*. *Proc. Natl. Acad. Sci.* **2019**, *116*, 3171–3176. DOI: 10.1073/pnas.1815661116.

(10) Lin, M. T.; Fukazawa, R.; Miyajima-Nakano, Y.; Matsushita, S.; Choi, S. K.; Iwasaki, T.; Gennis, R. B. *Escherichia coli* auxotroph host strains for amino acid-selective isotope labeling of recombinant proteins. *Methods. Enzymol.* **2015**, *565*, 45–66. DOI: 10.1016/bs.mie.2015.05.012.

(11) Gibson, D. G.; Young, L.; Chuang, R.-Y.; Venter, J. C.; Hutchison, C. A.; Smith, H. O. Enzymatic assembly of DNA molecules up to several hundred kilobases. *Nat. Methods* **2009**, *6* (5), 343–345. DOI: 10.1038/nmeth.1318.

(12) Schultz, E. E.; Braffman, N. R.; Luescher, M. U.; Hager, H. H.; Balskus, E. P. Biocatalytic Friedel–Crafts alkylation using a promiscuous biosynthetic enzyme. *Angew. Chem., Int. Ed. Engl.* **2019**, *58* (10), 3151–3155. DOI: 10.1002/anie.201814016.

(13) Gasteiger, E.; Hoogland, C.; Gattiker, A.; Duvaud, S. E.; Wilkins, M. R.; Appel, R. D.; Bairoch, A. Protein identification and analysis tools on the ExPASy server. In *The Proteomics Protocols Handbook*, Humana Press, 2005; pp 571–607.

(14) Lanz, N. D.; Grove, T. L.; Gogonea, C. B.; Lee, K.-H.; Krebs, C.; Booker, S. J. RlmN and AtsB as models for the overproduction and characterization of radical SAM proteins. *Methods. Enzymol.* **2012**, *516*, 125–152. DOI: 10.1016/B978-0-12-394291-3.00030-7.

(15) Goldman, S. A.; Bruno, G. V.; Polnaszek, C. F.; Freed, J. H. An ESR study of anisotropic rotational reorientation and slow tumbling in liquid and frozen media. *J. Chem. Phys.* **1972**, *56* (2), 716–735. DOI: 10.1063/1.1677222.

(16) Stoll, S.; Schweiger, A. EasySpin, a comprehensive software package for spectral simulation and analysis in EPR. *J. Magn. Reson.* **2006**, *178*, 42–55. DOI: 10.1016/j.jmr.2005.08.013.

(17) Hamoodi Murib, J.; Ritter, D. M.; Hamoodi Murib, B. J. Decomposition of nitrosyl disulfonate ion. I. Products and mechanism of color fading in acid solution. *J. Am. Chem. Soc.* **1952**, *74* (13), 3394–3398.

(18) Werst, M. M.; Davoust, C. E.; Hoffman, B. M. Ligand spin densities in blue copper proteins by Q-band ^{1}H and ^{14}N ENDOR spectroscopy. *J. Am. Chem. Soc.* **2002**, *113* (5), 1533–1538. DOI: 10.1021/ja00005a011.
(19) Deorowicz, S.; Debudaj-Grabysz, A.; Gudyś, A. FAMSA: Fast and accurate multiple sequence alignment of huge protein families. *Sci. Rep.* **2016**, *6* (1), 33964. DOI: 10.1038/srep33964.
(20) Gerlt, J. A.; Bouvier, J. T.; Davidson, D. B.; Imker, H. J.; Sadkhin, B.; Slater, D. R.; Whalen, K. L. Enzyme Function Initiative-Enzyme Similarity Tool (EFI-EST): a web tool for generating protein sequence similarity networks. *Biochim. Biophys. Acta. Proteins Proteom.* **2015**, *1854* (8), 1019–1037. DOI: 10.1016/j.bbapap.2015.04.015.
(21) Eddy, S. R. Accelerated profile HMM searches. *PLoS Comput. Biol.* **2011**, *7* (10), e1002195. DOI: 10.1371/journal.pcbi.1002195.
(22) Kalyaanamoorthy, S.; Minh, B. Q.; Wong, T. K. F.; von Haeseler, A.; Jermiin, L. S. ModelFinder: fast model selection for accurate phylogenetic estimates. *Nat. Methods.* **2017**, *14* (6), 587–589. DOI: 10.1038/nmeth.4285.
(23) Hoang, D. T.; Chernomor, O.; von Haeseler, A.; Minh, B. Q.; Vinh, L. S. UFBoot2: improving the ultrafast bootstrap approximation. *Mol. Biol. Evol.* **2018**, *35* (2), 518–522. DOI: 10.1093/molbev/msx281.
(24) Waterhouse, A. M.; Procter, J. B.; Martin, D. M.; Clamp, M.; Barton, G. J. Jalview version 2–a multiple sequence alignment editor and analysis workbench. *Bioinformatics* **2009**, *25* (9), 1189–1191. DOI: 10.1093/bioinformatics/btp033.
(25) Jumper, J.; Evans, R.; Pritzel, A.; Green, T.; Figurnov, M.; Ronneberger, O.; Tunyasuvunakool, K.; Bates, R.; Žídek, A.; Potapenko, A.; et al. Highly accurate protein structure prediction with AlphaFold. *Nature* **2021**, *596* (7873), 583–589. DOI: 10.1038/s41586-021-03819-2.
(26) Mirdita, M.; Schütze, K.; Moriwaki, Y.; Heo, L.; Ovchinnikov, S.; Steinegger, M. ColabFold: making protein folding accessible to all. *Nat. Methods* **2022**, *19* (6), 679–682. DOI: 10.1038/s41592-022-01488-1.
(27) Buchfink, B.; Xie, C.; Huson, D. H. Fast and sensitive protein alignment using DIAMOND. *Nat. Methods* **2015**, *12* (1), 59–60. DOI: 10.1038/nmeth.3176.
(28) Nayfach, S.; Pollard, K. S. Average genome size estimation improves comparative metagenomics and sheds light on the functional ecology of the human microbiome. *Genome Biol.* **2015**, *16* (1), 51. DOI: 10.1186/s13059-015-0611-7.
